# Transcriptome sequencing and microarray design for functional genomics in the extremophile *Arabidopsis* relative *Thellungiella salsuginea* (*Eutrema salsugineum*)

**DOI:** 10.1186/1471-2164-14-793

**Published:** 2013-11-14

**Authors:** Yang Ping Lee, Federico M Giorgi, Marc Lohse, Kotryna Kvederaviciute, Sven Klages, Björn Usadel, Irute Meskiene, Richard Reinhardt, Dirk K Hincha

**Affiliations:** 1 Max-Planck-Institut für Molekulare Pflanzenphysiologie, Am Mühlenberg 1, D-14476 Potsdam, Germany; 2 FELDA Agricultural Services Sdn Bhd, Tingkat 7, Balai Felda, Jalan Gurney 1, 54000 Kuala Lumpur, Malaysia; 3 Center for Computational Biology and Bioinformatics, Columbia University, 10032 New York, NY, USA; 4 Institute of Biotechnology, University of Vilnius, V. Graičiūno 8, LT-02241 Vilnius, Lithuania; 5 Max-Planck-Institute for Molecular Genetics, Ihnestrasse 63-73, D-14195 Berlin, Germany; 6 Institut für Biologie I, RWTH Aachen, Worringer Weg 1, D-52056 Aachen, Germany; 7 IBG-2: Pflanzenwissenschaften, Forschungszentrum Jülich, D-52425 Jülich, Germany; 8 Max F. Perutz Laboratories, University and Medical University of Vienna, Dr. Bohrgasse 9, A-1030 Vienna, Austria

**Keywords:** *Arabidopsis thaliana*, Cold acclimation, Gene annotation, LEA proteins, MAP kinases, Microarray design, microRNAs, Protein phosphatases, *Thellungiella salsuginea*, Transcriptome sequencing

## Abstract

**Background:**

Most molecular studies of plant stress tolerance have been performed with *Arabidopsis thaliana*, although it is not particularly stress tolerant and may lack protective mechanisms required to survive extreme environmental conditions. *Thellungiella salsuginea* has attracted interest as an alternative plant model species with high tolerance of various abiotic stresses. While the *T. salsuginea* genome has recently been sequenced, its annotation is still incomplete and transcriptomic information is scarce. In addition, functional genomics investigations in this species are severely hampered by a lack of affordable tools for genome-wide gene expression studies.

**Results:**

Here, we report the results of *Thellungiella de novo* transcriptome assembly and annotation based on 454 pyrosequencing and development and validation of a *T. salsuginea* microarray. ESTs were generated from a non-normalized and a normalized library synthesized from RNA pooled from samples covering different tissues and abiotic stress conditions. Both libraries yielded partially unique sequences, indicating their necessity to obtain comprehensive transcriptome coverage. More than 1 million sequence reads were assembled into 42,810 unigenes, approximately 50% of which could be functionally annotated. These unigenes were compared to all available *Thellungiella* genome sequence information. In addition, the groups of Late Embryogenesis Abundant (LEA) proteins, Mitogen Activated Protein (MAP) kinases and protein phosphatases were annotated in detail. We also predicted the target genes for 384 putative miRNAs. From the sequence information, we constructed a 44 k Agilent oligonucleotide microarray. Comparison of same-species and cross-species hybridization results showed superior performance of the newly designed array for *T. salsuginea* samples. The developed microarrays were used to investigate transcriptional responses of *T. salsuginea* and *Arabidopsis* during cold acclimation using the MapMan software.

**Conclusions:**

This study provides the first comprehensive transcriptome information for the extremophile *Arabidopsis* relative *T. salsuginea*. The data constitute a more than three-fold increase in the number of publicly available unigene sequences and will greatly facilitate genome annotation. In addition, we have designed and validated the first genome-wide microarray for *T. salsuginea*, which will be commercially available. Together with the publicly available MapMan software this will become an important tool for functional genomics of plant stress tolerance.

## Background

The majority of molecular studies of plant stress tolerance have been performed with *Arabidopsis thaliana*, although it is not an extremophile and can therefore be expected to lack many protective mechanisms required to survive extreme environmental conditions. *Thellungiella salsuginea* has therefore attracted increasing interest as an alternative plant model species that possesses the characteristics of an extremophile, i.e. high tolerance of salinity, freezing, nitrogen-deficiency and drought stress [1-7]. The genus *Thellungiella* is part of the *Brassicaceae* family and therefore related to *Arabidopsis*[8,9]. The species name has sometimes been used synonymously with *Thellungiella halophila*, but it is clear now that three distinct *Thellungiella* species (*T. halophila*, *T. salsuginea*, and *T. botschantzevii*) have to be distinguished [10]. *T. salsuginea* is also referred to as *Eutrema salsugineum*, however, we prefer to stay with the older and better established species name as that is not in conflict with the taxonomy [10]. On the other hand, *Thellungiella parvula* is not as closely related to these other species as previously thought and should be called *Schrenkiella parvula*[10]. However, to avoid unnecessary confusion in the assignment of sequence information, we have used the species names used in the respective publications in all comparisons with our data.

*T. salsuginea* resembles *Arabidopsis* in many important features such as short life cycle, self-fertility and the possibility to transform plants by the floral-dip method [4]. While the genomes of *T. salsuginea*[11,12] and *T. parvula*[13] have recently been sequenced, important functional genomics tools such as dedicated microarrays are still lacking for these species.

*Arabidopsis*-based microarrays [2,4,14-17] and a cDNA array comprising 3,628 unique *T. salsuginea* ESTs [18] have been used to profile the effects of abiotic stress conditions on the *T. salsuginea* transcriptome. However, *T. salsuginea* is not in the *Arabidopsis* genus and therefore has several distinguishing genetic and developmental properties [1,4]. Identity between the *Arabidopsis* genome sequence and *T. salsuginea* ESTs (Expressed Sequence Tags) is between 95% and 60%, but there are also specific ESTs in *T. salsuginea*[4,7,19,20]. Importantly, analysis of *T. salsuginea* mRNA using the Affymetrix *Arabidopsis* GeneChip ATH1 produced signals significantly above background for only 20% of the probe sets [17], indicating the necessity for a dedicated *T. salsuginea* microarray for cost-effective transcriptome studies.

Transcriptome sequencing is playing an increasingly important role in functional genomic studies of organisms without full genome sequences (e.g. [21-25]). The 454 technology is an efficient and cost effective way to generate high-throughput EST data for such organisms [26-30], providing the possibility for *de novo* assembly of transcriptomes from uncharacterized genomes [31,32].

Large scale isolation of ESTs is a powerful approach for the discovery of novel genes, the investigation of genes of unknown function, comparative genomic studies and identification of exon/intron boundaries [33]. There are currently 44,559 *T. salsuginea* EST sequences in the GenBank dbEST (as of June 19, 2012; [34]) and 19,429 full-length cDNAs are available from RIKEN [35]. These numbers are far smaller than for *Arabidopsis* (1,529,700 ESTs as of June 19, 2012), indicating the need for further gene discovery and characterization in *T. salsuginea*.

Here, we have sequenced, assembled and annotated the transcriptome of the *T. salsuginea* Yukon accession using 454 pyrosequencing. On the basis of these transcriptome sequences, we have developed a dedicated *T. salsuginea* microarray using the Agilent long oligonucleotide array platform. This array has been benchmarked against the corresponding *Arabidopsis* array and tested in comparative cold acclimation experiments [6].

## Results

### 454 sequencing and transcriptome assembly

**Figure 1:**
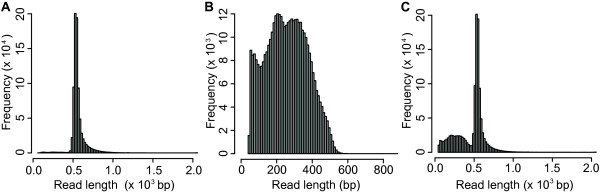
**Expressed sequence tags of *T. salsuginea *generated using 454 pyrosequencing.** Frequency distribution of 454 read lengths derived from **(A)** non-normalized and **(B)** normalized library, and **(C)** combined data from both libraries.

We constructed non-normalized and normalized cDNA libraries of *T. salsuginea* from various tissues derived from soil- and hydroponically-grown plants, including rosette and cauline leaves, roots, flowers, and siliques of plants treated with cold, drought and salt to achieve comprehensive transcriptome information. Using the GS FLX 454 sequencer, we generated 400,631 and 811,683 reads from a normalized and non-normalized library, respectively, comprising 562.67 Mbp with an average read length of 464 bp (Table 1). The average read length was greater for ESTs derived from the non-normalized library (566 bp) than from the normalized library (257 bp) and the proportion of reads shorter than 257 bp was larger for the normalized library compared to the non-normalized library (Figure 1), as a result of cDNA fragmentation in the procedure for constructing normalized libraries.

**Table 1:** **Summary of *T. salsuginea *** **ESTs generated from 454 sequencing and assembled contigs**

**Library**	**Non-normalized**	**Normalized**	**Combined**^**1**^
Number of reads	811,683	400,631	1,212,314
Number of contaminated reads^2^	4,496	2,506	7,002
Average read length (bp)	566	257	464
Total reads (Mbp)	459.54	103.13	562.67
Number of assembled reads	712,262	376,509	1,060,666
Number of contigs^3^	33,870	28,928	46,220
Number of contigs with only 454 reads^4^	21,662	16,949	33,147
Average coverage	11.99	6.74	12.63
N50 (bp)	665	632	646
Average contig length (bp)	621	502	567
Number of contigs with ORF^5^	33,625	28,416	45,583
Number of *Arabidopsis* peptides found^6^	23,787	23,220	24,457

^1^Combined 454 reads derived from normalized and non-normalized library.

^2^Best hits with E-value < 1e-10, non streptophyta sequences (NCBI, nr, nt, as of February 12, 2010).

^3^Includes publicly available *T. salsuginea* ESTs (as of April 19, 2010).

^4^Number of contigs minus number of contigs assembled with at least one publicly available EST.

^5^Open reading frames (ORFs) predicted including partial ORFs (Min et al. [83]).

^6^Any hits with E-value < 0.001 from BLASTX search against TAIR9 database (27,379 peptides).

**Figure 2:**
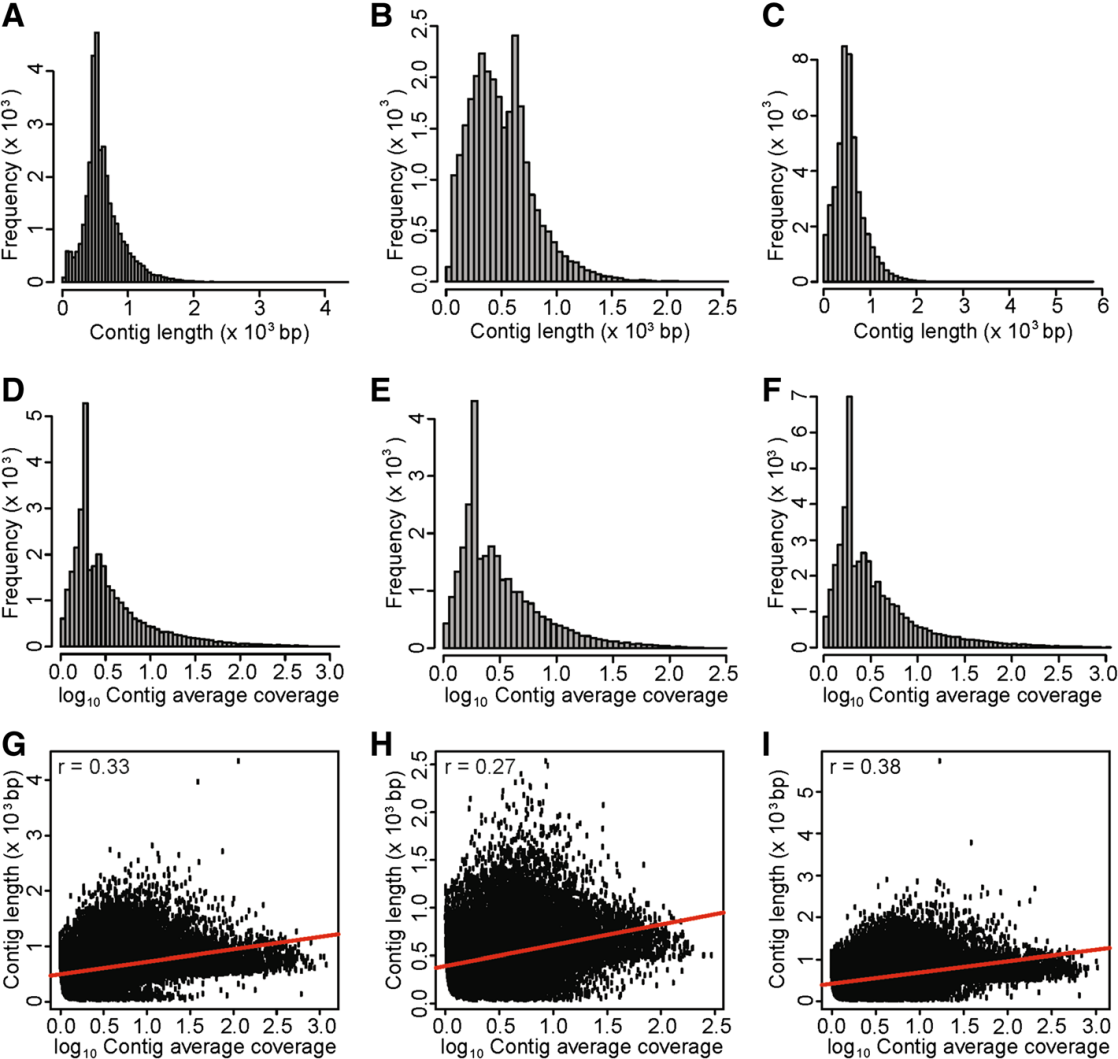
**Size distribution and characteristics of assembled contigs.** Frequency distribution of contigs assembled from the pool of publicly available *T. salsuginea* ESTs and 454 reads derived from **(A)** non-normalized library, **(B)** normalized library or **(C)** combined libraries. Frequency distribution of contig average coverage for the assembly of publicly available *T. salsuginea* ESTs pooled with 454 reads derived from **(D)** non-normalized library, **(E)** normalized library or **(F)** combined libraries. Pearson’s correlations between contig length and contig average coverage for the assembly of publicly available *T. salsuginea* ESTs pooled with 454 reads derived from **(G)** non-normalized library, **(H)** normalized library or **(I)** combined libraries.

We combined the 454 reads with 44,551 publicly available *T. salsuginea* ESTs and performed a transcriptome assembly with MIRA [36]. This *de-novo* assembly of the combined libraries generated 46,220 contigs with an average length of 567 bp (Table 1, Figure 2C). The contigs showed a similar trend of frequency distribution of average contig coverage (reads per contig length) for three different assemblies (Figure 2D, E, F). Pearson correlation analysis revealed a significant positive correlation (*p* < 10^-6^) between contig length and contig average coverage (Figure 2G, H and I). This result suggests that the consensus nucleotide sequences of long contigs are confidently supported with more reads than the shorter contigs. The assembly of the combined reads increased the number of contigs and average coverage (12.63 reads per nucleotide in the template), but the average contig length was smaller when compared to the non-normalized library (Table 1, Figure 2A and C). Of 46,220 contigs, 33,147 (71.7%) were unique and had not been reported before.

**Figure 3:**
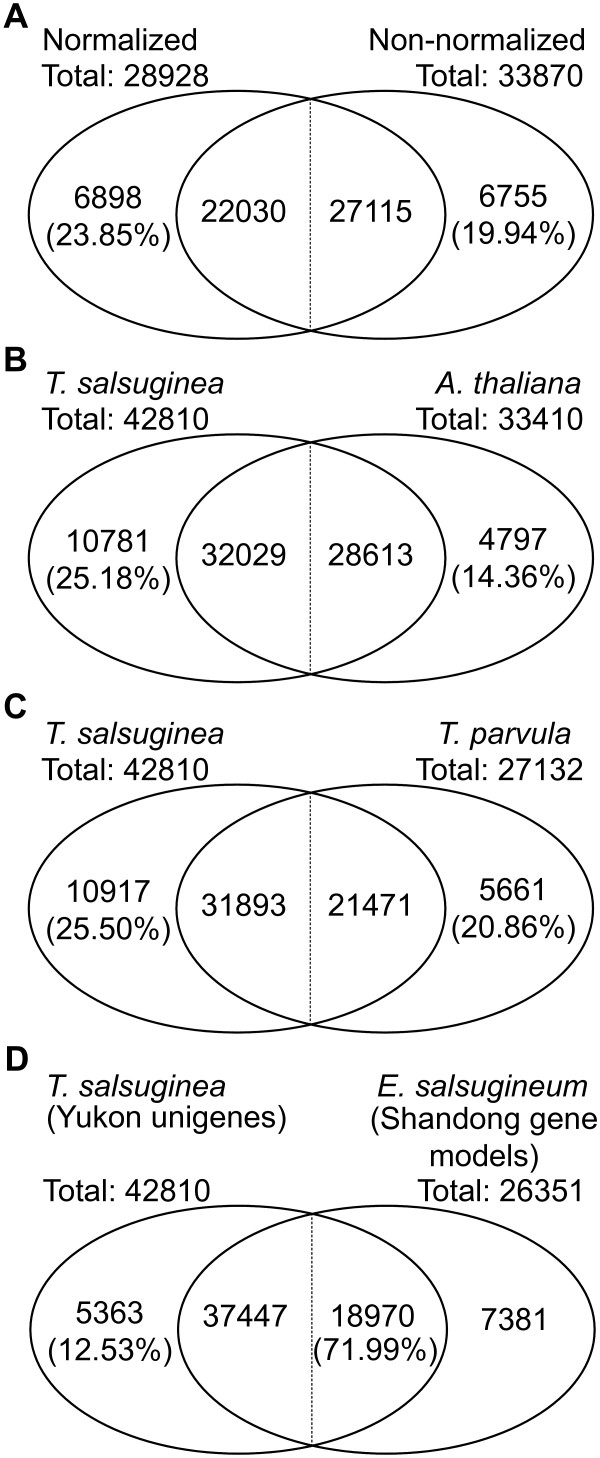
**Similarity of *T. salsuginea *(Yukon) unigenes to *Arabidopsis *and *T. parvula *orthologs and *T. salsuginea/Eutrema salsugineum *(Shandong) gene models.** Venn diagrams depicting the number of unique unigenes derived only from **(A)** normalized or non-normalized libraries, **(B)***T. salsuginea* or *Arabidopsis,***(C)***T. salsuginea* or *T. parvula* (*Schrenkiella parvula*) orthologs, and *T. salsuginea* (*Eutrema salsugineum*) Yukon uniges or Shandong gene models **(D)**. Vertical dividing lines indicate non-matched overlap values for both libraries and species due to the nature of the BLAST search algorithm.

We further examined whether sequencing both a normalized and a non-normalized library increased the number of unique transcripts that were detected. The normalization procedure produced about 24% (6898) unique contigs that were not found in the non-normalized library, while the non-normalized library yielded about 20% (6755) unique contigs using BLASTN with E value threshold of 10^-50^. This indicates that to approach full transcriptome information, the sequencing of both types of libraries is necessary. No consensus number was found for the matched contigs between the two libraries using BLASTN in reciprocal searches (Figure 3A).

To assess the quality of the assembly, we predicted open reading frames (ORFs) of the contigs and used BLASTX to align all contigs to the TAIR9 *Arabidopsis* annotated protein database using an E-value threshold of 10^-3^. Of 27,379 *Arabidopsis* peptides, 24,457 unique peptides were matched to contigs generated from the combined libraries (Table 1), indicating a high quality of the transcriptome assembly.

### Gene discovery

The transcriptome assembly yielded 4,020 contigs sharing more than 99% sequence identity and coverage with at least one other contig in the assembly, likely constituting alleles of the same genes (or very close paralogs) with single nucleotide polymorphysms (SNPs). Because the seed material was not derived from single seed descent, the plants used in the experiments were not genetically identical, making the presence of SNPs in this plant population likely. We therefore condensed these 4,020 contigs into 610 clusters with ambiguous nucleotides at the SNP sites, reducing the final number of unigenes to 42,810. Since *T. salsuginea* and *Arabidopsis* are both members of the *Brassicaceae* family, we estimated the number of *T. salsuginea* unigenes that were orthologous to *Arabidopsis* genes using BLASTX. This identified 32,029 unigenes as orthologs of *Arabidopsis* genes, while approximately 25% were unique to *T. salsuginea*. No consensus overlap was found for the number of matched sequences between both species when a reciprocal search was performed, because every given reference sequence may hit more than one paralog in the other species, while also more than one homologous sequence in one species may hit a single sequence in the other species (Figure 3B). The genome sequence of *T. parvula* was published recently [13] and although *T. salsuginea* and *T. parvula* are related, about 26% of *T. salsuginea* unigenes have no counterparts in *T. parvula*, while 21% of the *T. parvula* genes have no orthologs in *T. salsuginea* (Figure 3C), probably reflecting the fact that the relationship between *T. salsuginea* and *T. parvula* (*Schrenkiella parvula*[10]) is not as close as originally assumed. On the other hand, approximately 88% (37,622 of 42,810) of the *T. salsuginea* unigenes could be aligned to 3,703 *T. salsuginea* genomic contig sequences comprising 135.6 Mbp of genome sequence [11] using BLASTN with a stringent threshold (E-value < 10^-50^).

More recently the *T. salsuginea* (Shandong accession) genome sequence obtained by Sanger sequencing technology has been published [12]. We estimated that 72% (18,970 out of 26,351) of the gene models predicted from the genome were expressed under our experimental conditions (BLASTN, E-value < 10^-10^). On the other hand, 37,447 *T. salsuginea* Yukon unigenes were best matched (BLASTN, E-value < 10^-10^) with the Shandong gene models (Figure 3D). Therefore, 5,363 unigenes from the Yukon accession have no counterpart among the predicted gene models from the Shandong accession and may be Yukon-specific transcripts (Figure 3D). Approximately 56.7% of matched unigenes (21,228 out of 37,447) were estimated to be full length in relation to the published gene models from the genome sequence (E-value <10^-10^ and total query coverage > 90%).

We further investigated how many Yukon unigenes were likely derived from gene duplication events in relation to Shandong gene models. We assessed the sequence relationships between Yukon accession unigenes and Shandong accession gene models by BLASTN analysis (E-value < 10^-10^ and query coverage >90%). Based on these criteria (21,228 Yukon unigenes matching Shandong gene models), we assessed whether the relationships were one-to-one (single copy number conserved in both accessions), one-to-many (one Yukon transcript matches more than one Shandong gene model), many-to-one (more than one Yukon transcript uniquely matches a single Shandong gene model), or many-to-many (larger homologous groups with several members from both accessions). 376 putatively duplicated Yukon unigenes were identified in relation to Shandong gene models. These unigenes were enriched with genes involved in protein synthesis, nucleotide metabolism, DNA synthesis, light reaction of photosynthesis, regulation of transcription, redox and storage proteins (Table 2).

**Table 2:** Enrichment of unigenes in MapMan terms for 12,272 duplicated Yukon unigenes

**Mapman bin code**	**Mapman term**	**Number of duplicated Yukon unigenes**	Fisher’s exact test *P*-value (Bonferroni-corrected)*
29.2.1	protein.synthesis.ribosomal protein	558	1.48E-18
11.1	lipid metabolism – Fatty Acid synthesis and Fatty Acid elongation	89	1.56E-06
29.5	protein.degradation	652	3.28E-05
34	transport	433	1.25E-04
30	signalling	488	2.93E-04
1.1.1.01	PS.lightreaction.photosystem II.LHC-II	52	3.98E-04
20.1.7	stress.biotic.PR-proteins	29	1.00E-03
20.1	stress.biotic	93	1.86E-03
29.3.4	protein.targeting.secretory pathway	95	3.52E-03
1.1.2.01	PS.lightreaction.photosystem I.LHC-I	41	4.32E-03
23.4.010	nucleotide metabolism.phosphotransfer and pyrophosphatases.nucleoside diphosphate kinase	21	8.96E-03
20.2.3	stress.abiotic.drought/salt	63	1.17E-02
30.3	signalling.calcium	129	1.20E-02
21	redox.regulation	167	2.28E-02
11.1.012	lipid metabolism.FA synthesis and FA elongation.ACP proteins	18	2.49E-02
17.6.3	hormone metabolism.gibberelin.induced-regulated-responsive-activated	18	2.49E-02
27.3.25	RNA.regulation of transcription.MYB domain transcription factor family	14	2.54E-02
27.3.52	RNA.regulation of transcription.Global transcription factor group	16	2.70E-02
29.1	protein.aa activation	59	2.72E-02
4.02	glycolysis.PGM	10	3.22E-02

*The Bonferroni-corrected Fisher’s exact test P-value that was extracted from MapMan term enrichment analysis using MEFISTO software [101].

The single nucleotide polymorphisms (SNPs) between Shandong gene models and Yukon unigenes were called using VarScan v2.3.5 [37] as allelic variation may contribute to the variation of gene expression and the phenotypes of the accessions [38-40]. Using Shandong gene models as reference sequences, we identified 16,209 putative non-ambiguous SNPs between Yukon and Shandong in 4,020 Shandong gene models (Table 3, Additional file 1). Whether the identified SNPs contribute to differences in gene expression and/or phenotypic variation, e.g. in stress responses, remains to be investigated.

**Table 3:** Non-ambiguous SNPs identified between Shandong gene models and Yukon unigenes

SNP frequency*	**Number of Shandong gene models**
1	1,119
2	854
3	590
4	374
5	302
6	179
7	122
8	101
9	69
≥ 10	310
Total	4,020

*Detected nonambiguous SNPs between Shandong gene models and Yukon unigenes. The Yukon unigenes were aligned to the reference sequence of the Shandong gene models and called for variations using VarScan v2.3.5 [37].

### Functional annotation

**Figure 4:**
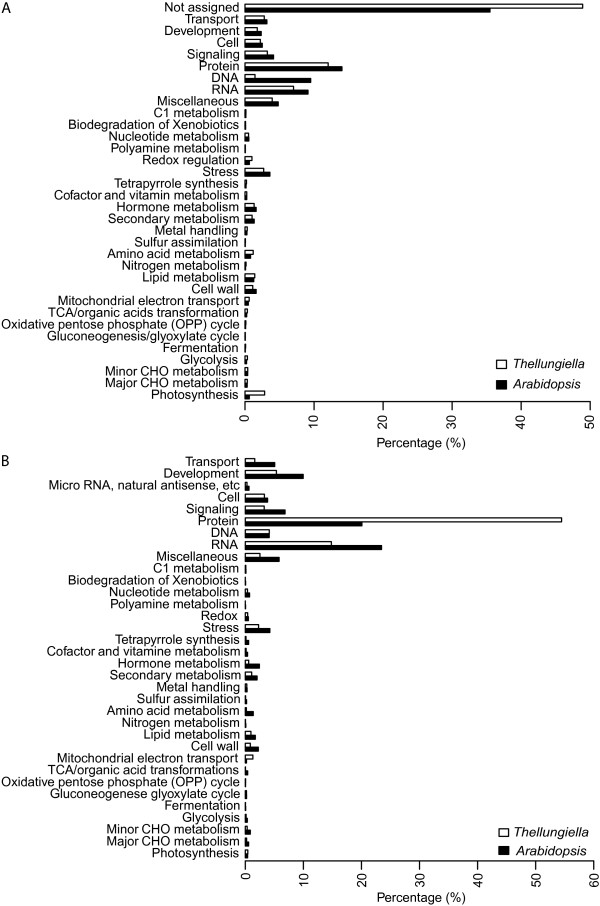
**Distribution of predicted *T. salsuginea *gene functions in comparison to *Arabidopsis thaliana*. (A)** Functional classes of encoded proteins and **(B)** micro RNA prediction and distribution of their target genes.

Sequence-based functional annotation of the 42,810 unigenes was performed using Mercator [41] and MapMan ontology [42] to predict and classify the protein coding unigenes. The Mercator tool predicts the function of input DNA sequences by searching a variety of reference databases (currently six are available: three BLAST-based, two RPSBLAST-based and InterProScan). Subsequently, each input gene is assigned to one or more functional MapMan bins based on the manually curated binning of the reference database entries [42,43]. We compared the distribution of gene functions and found that a large proportion of *Arabidopsis* (35.5%) and *T. salsuginea* unigenes (48.9%) could not be classified to any functional bin (Figure 4A). A large number of genes with known functions were classified in bins of protein, DNA, RNA, signaling, transport, stress, cell, development, photosynthesis and miscellaneous for *Arabidopsis* (53.8%) and *T. salsuginea* (40.0%). The distribution of gene functions was similar in both species with similar proportions of genes for each bin except gene functions related to DNA metabolism (9.5% in *Arabidopsis* and 1.5% in *T. salsuginea*) and differences of about 2% between *Arabidopsis* and *T. salsuginea* in bins for RNA, protein and photosynthesis (Figure 4A).

### microRNA prediction

Higher plant transcriptomes contain a large number of non-coding RNAs including microRNA (miRNA) that function in diverse regulatory processes that influence e.g. growth and development [44]. Since primary miRNA transcripts are polyadenylated [45-47] it seemed possible that the *T. salsuginea* unigenes contain primary miRNA transcripts that code for one or more miRNA stem loops that could be further processed to mature miRNAs. We scanned all unigenes using BLASTN with a threshold E-value of 10^-10^ against the complete set of miRNA precursor transcripts from the plant miRNA database [48] and obtained 384 unigenes that putatively encode miRNA precursor transcripts. The predicted target transcripts of the corresponding miRNAs were classified according to their functions using MapMan ontology and the predicted targets were compared with those of *Arabidopsis* miRNAs (Figure 4B). The proportion of predicted targets in the different functional classes was generally lower for *T. salsuginea* than for *Arabidopsis* except for target functions related to protein metabolism (54.5% vs. 20.1%) and mitochondrial electron transport (1.3% vs. 0.2%), where *T. salsuginea* showed higher proportions compared to *Arabidopsis*.

### Genes encoding protein kinases and protein phosphatases

Mitogen activated protein kinases (MAPK) and MAPK phosphatases are important components that mediate abiotic stress signaling in plant cells [49]. Members of the families of protein tyrosine phosphatases (PTPs), including dual specificity phosphatases (DSPs) and protein phosphatases 2C (PP2Cs) have been characterized as plant MAPK phosphatases that regulate stress responses [50,51]. In the PP2C gene family the A-type PP2C phosphatases are generally upregulated in response to abscisic acid (ABA), whereas B-type PP2Cs (AP2Cs) have been characterized as MAPK phosphatases [51]. Since drought or salt stress induces ABA biosynthesis in plants and MAPK cascades have been related to salt stress tolerance [49], it was of high interest to identify putative orthologous proteins of PP2Cs and PTPs/DSPs, as well as MAPKs and MAPK kinases (MAPKKs) in the extremophile *T. salsuginea.*

Individual *T. salsuginea* putative MAPKK, MAPK, PP2C, DSP and PTP proteins were designated according to the corresponding orthologous protein annotations in *Arabidopsis* (Additional files 2, 3 and 4) and their phylogenetic relationships were visualized in unrooted phylogenetic trees (Additional files 5, 6 and 7). Using 80 PP2Cs from *Arabidopsis*[51-53] as well as 74 and 75 PP2Cs encoded in the *T. salsuginea* and *T. parvula* genomes [11-13], respectively, led to the identification of 118 PP2C unigenes in the sequenced *T. salsuginea* ESTs that could be translated into 59 PP2C proteins (Table 4, Additional file 8).

**Table 4:** **Number of putative protein tyrosine phosphatase (PTP), dual specificity phosphatase (DSP), protein phosphatase 2C (PP2C), mitogen activated protein kinase (MAPK) and MAPK kinase (MAPKK) unigenes and proteins in the *T. salsuginea *** **( *T.s. *), *T. parvula *** **( *T.p. *) and *A. thaliana *** **( *A.t. *) genomes**

	**Unigenes**	**Proteins from unigenes**	**Proteins from genome**
**PTP *T.s.***	1	1	1
**PTP *T.p.***	na	na	2
**PTP *A.t.***	na	na	1
**DSP *T.s.***	28	18	23
**DSP *T.p.***	na	na	20
**DSP *A.t.***	na	na	22
**PP2C *T.s.***	118	59	75
**PP2C *T.p.***	na	na	74
**PP2C *A.t.***	na	na	80
**MAPK *T.s.***	25	13	18
**MAPK *T.p.***	na	na	19
**MAPK *A.t.***	na	na	20
**MAPKK *T.s.***	8	6	10
**MAPKK *T.p.***	na	na	11
**MAPKK *A.t.***	na	na	10

na = not applicable.

All plant species studied so far revealed a single gene encoding a PTP [54]. Correspondingly, a single unigene of a PTP was identified in the *T. salsuginea* transcriptome. However, two PTP genes were identified in the genome of *T. parvula* (Table 4). Reciprocal BLAST of 22 and 20 DSP sequences from *Arabidopsis*[54] and *T. parvula*[13], respectively, revealed 28 DSP encoding unigenes (Table 4) that code for 18 *T. salsuginea* DSP proteins (Additional file 8).

The genome sequences of *T. salsuginea* and *T. parvula*[11-13] revealed 18 and 19 MAPK encoding genes, respectively, in comparison to 20 MAPKs [55] found in *Arabidopsis*. Here, analysis of MAPKs in the transcriptome of *T. salsuginea* identified 25 unigenes, which were translated into 13 putative MAPK proteins (Table 4, Additional file 8). MAPKK proteins are represented by 10 and 11 members in the genomes of *Arabidopis*[55] and *T. parvula*[13], respectively. In the *T. salsuginea* trancriptome we identified eight ESTs representing MAPKK, corresponding to six MAPKK proteins (Table 4, Additional file 8).

### Genes encoding Late Embryogenesis Abundant (LEA) proteins

**Figure 5:**
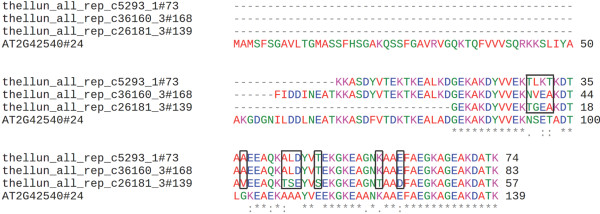
**Multiple sequence alignment of COR15A orthologous proteins between *T. salsuginea *and *Arabidopsis*.** Boxes indicate substitutions of amino acids among *T. salsuginea* COR15A orthologous proteins. Identical amino acids are marked with a star. Amino acids showing conserved substitutions are marked with two dots, and semi-conserved substitutions are marked with a single dot.

The accumulation of LEA proteins (or their transcripts) has been associated with environmental stress conditions such as cold, drought, or desiccation in plants, as well as in some bacteria and invertebrates [56,57]. In *Arabidopsis* 51 genes encoding LEA proteins have been identified and all genes were shown to be expressed in some tissue and/or under particular stress conditions [58]. Most of the *Arabidopsis* LEA proteins were predicted to be highly hydrophilic, intrinsically disordered proteins. Since LEA proteins are generally assumed to play important roles in cellular stress tolerance, it was of interest to identify potential orthologous proteins in the extremophile *T. salsuginea*. Therefore, the 51 *Arabidopsis* LEA proteins were reciprocally compared by BLASTX (threshold E-value 10^-10^) with the translated unigenes. This resulted in 148 putative *T. salsuginea* LEA protein sequences (Additional file 9). They were aligned with the *Arabidopsis* LEA proteins using ClustalW and an unrooted dendogram was drawn (Additional file 10) assigning the putative LEA proteins to the corresponding Pfam domain/motif groups (Additional file 11). Due to high similarity at the protein sequence level, these proteins were condensed to 52 protein sequences that showed distinct models (Additional file 11). For example, one of the best characterized *Arabidopsis* LEA proteins is COR15A (LEA24) which clustered with three orthologous *T. salsuginea* proteins (73, 139, 168, Additional file 10) encoded by distinct unigenes. However, multiple protein sequence alignment showed that amino acid sequences translated from thellun_all_rep_c5293 (73) and thellun_all_rep_c26181 (139) were highly similar to thellun_all_rep_c36160 (168; Figure 5) which is the longest of the three translated polypeptides. We therefore chose translated frame +3 of thellun_all_rep_c36160 as the representative COR15A orthologous protein in *T. salsuginea* (Additional file 12).

Following this strategy for all 148 putative *T. salsuginea* LEA proteins identified 15 *Arabidopsis* LEA proteins without an orthologous protein in the *T. salsuginea* transcriptome. However, a BLASTN search (E < 1e-50) revealed the presence of genes encoding 12 of these proteins in the *T. salsuginea* genome. For example, there were no apparent orthologs of LEA_5 proteins among the unigenes, but the BLASTN search of the gene sequences (At2g40170 and At3g51810) against the *T. salsuginea* genome retrieved two genomic sequences (AHIU01008450.1 and AHIU01008580.1), indicating that two LEA_5 proteins are encoded in the *T. salsuginea* genome, but probably not expressed under our experimental conditions. LEA_4 and dehydrin are the two largest groups of LEA proteins in *Arabidopsis* and this was also true for *T. salsuginea* with 18 LEA_4 proteins in both species. Interestingly, *T. salsuginea* contains more dehydrins (17 vs 10) than *Arabidopsis*. This is due to the fact that some *Arabidopsis* LEA proteins were represented by several *T. salsuginea* orthologs. For instance, LEA23 (At2g42530; COR15B) and LEA51 (At5g66400; RAB18) were each represented by four distinct protein sequences and several others by three (Additional file 12).

Overall, the majority of putative *T. salsuginea* LEA proteins showed negative GRAVY (grand average of hydropathy) values indicating the expected high hydrophilicity (Additional file 12). Since a precise *in-vivo* function has not been established for any *Arabidopsis* LEA protein, it is currently impossible to evaluate the functional significance of the multiple orthologous *T. salsuginea* proteins.

### Design and performance of a dedicated T. salsuginea microarray

Between 2 and 12 60-mer oligonucleotide probes were designed for each *T. salsuginea* unigene, depending on the length of the predicted gene. With this oligonucleotide sequence information as input, 400 K arrays were produced by Agilent. A Pre-Selection Strategy (PSS) [59] was used to select the best-performing probes by hybridizing either cRNA derived from pooled total RNA (identical to the RNA used in cDNA library construction for sequencing) or genomic DNA to these arrays. On the basis of the hybridization signal strength, the 43,800 best-performing probes were selected. 40,952 probes were selected on the basis of their hybridization signals with the cRNA sample and 2,848 additional probes were derived from the hybridization with genomic DNA. Using the Agilent eArray Platform [60], 44 K arrays were produced from these oligonucleotide sequences. For 2,237 unigenes, the number of features on the array allowed the design of two different probes for the same contig, while for the remaining 39,326 unigenes only one probe was designed. The resulting 43,800 probes therefore represented 41,563 unigenes.

To test the newly designed arrays, they were hybridized with total RNA derived from control (NA) or 14-day cold acclimated (ACC) *T. salsuginea* plants from three independent biological experiments. Spot intensities between replicates were highly correlated with r-values of Pearson correlations between 0.985 and 0.993, *p* < 2.2 × 10^-16^ (Additional file 13). In addition, the log_2_-transformed fold change in hybridization signals between samples from non-acclimated and cold acclimated plants for the 2,237 pairs of probes targeting the same unigene were also highly correlated (Additional file 14, r = 0.951, p < 2.2 × 10^-16^). These results validate the probe design and selection with the PSS method.

**Figure 6:**
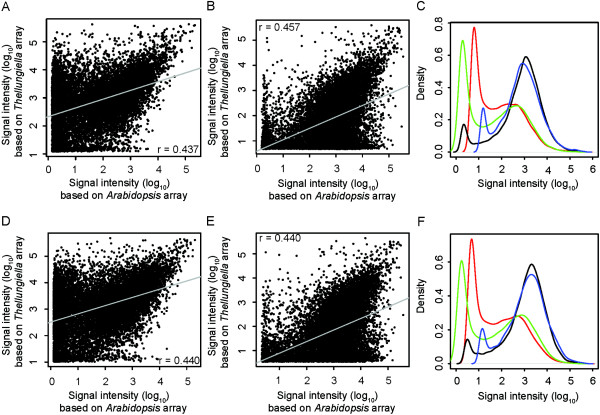
**Comparison of median raw signal intensities of 15,557 genes matched between *T. salsuginea *and *Arabidopsis *arrays.***T. salsuginea* genes were matched with *A. thaliana* genes using BLASTX, with a significance cut-off of E < 10^-10^ for a sequence similarity search against the *Arabidopsis* TAIR10 database. This resulted in 15,557 genes that matched between the two microarrays. Pearson’ s correlations (*p* < 2.2 × 10^-16^) are shown between log10-transformed signal intensities obtained from *T. salsuginea* and *Arabidopsis* arrays hybridized with identical leaf RNA samples from either non-acclimated Yukon **(A)** or Col-0 **(B)**, or cold acclimated Yukon **(D)** or Col-0 **(E)**. The median density signal distributions of all 15,557 probes after hybridization with non-acclimated **(C)** or cold acclimated RNA samples **(F)** are also shown. The green lines indicate the density distribution of median signal intensity of Yukon samples hybridized on the array designed for *Arabidopsis*, while the blue lines show the density distribution of Yukon samples hybridized on the array designed for *T. salsuginea*. The red lines show the density distribution of Col-0 samples hybridized on the Agilent array designed for *T. salsuginea* and the black lines show the density distribution of Col-0 samples hybridized on the array designed for *Arabidopsis*.

To further examine the quality of the *T. salsuginea* array and to assess whether hybridization results from a dedicated array are superior to cross-species hybridization using the *Arabidopsis* array from the same manufacturer, we performed reciprocal array hybridizations. In this experiment, RNA from the Yukon accession of *T. salsuginea* and RNA from the Col-0 accession of *Arabidopsis* were both hybridized to the Agilent *T. salsuginea* and *Arabidopsis* arrays. For both species, RNA was extracted from acclimated and non-acclimated plants from three independent biological replicates. The comparison between the performance of the two arrays was limited to a set of probes that represent genes that are highly similar between the two species. These genes were identified using BLASTX with a threshold E-value of 10^-10^ that resulted in 15,557 orthologous genes. The signal intensities (median values) of the corresponding probes were significantly correlated (Figure 6A, B, D, E, r-values between 0.437 and 0.457, and *p* < 2.2 × 10^-16^) between the *T. salsuginea* and *Arabidopsis* arrays when hybridized with identical RNA samples. However, closer examination of these correlations revealed that a substantial proportion of probes yielded very low signal intensities in cross-species hybridizations. For example, when RNA from non-acclimated *T. salsuginea* plants was hybridized to *Arabidopsis* arrays (Figure 6A), this yielded 4,426 genes with log_10_ signal intensities below 1, while the log_10_ signal intensities were above 2 when identical RNA samples were hybridized to *T. salsuginea* arrays.

Significantly, the distribution of raw signal intensities from all probes on the arrays revealed a bimodal distribution [61,62] with a dominant density peak at high signal intensities (likely derived from perfect-match hybridizations) and a minor peak at low signal intensities (likely derived from noise and mismatch hybridization) for the same-species array hybridizations (black and blue lines, Figure 6C and F). In contrast, cross-species array hybridizations yielded predominantly low signal intensities (green and red lines, Figure 6C and F), suggesting that these hybridizations were dominated by non-specific signals.

**Figure 7:**
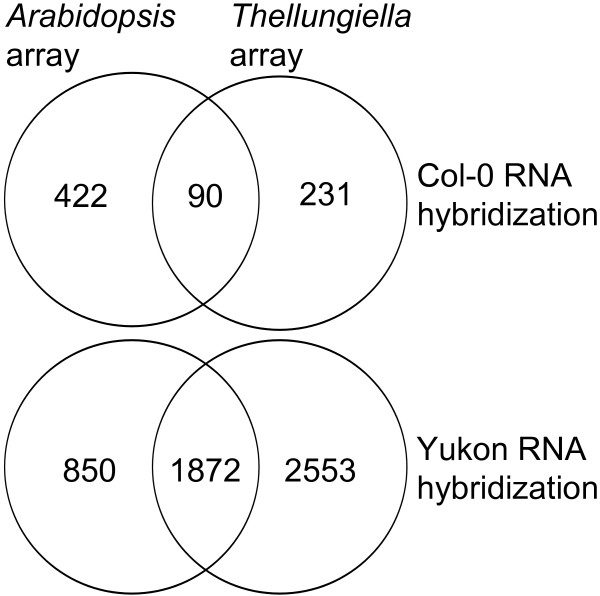
**Comparison of significant gene expression changes determined using the Agilent *Arabidopsis *or *Thellungiella salsuginea *arrays.** Venn diagrams show the overlap between genes with significant (linear model, FDR, *p* < 0.05) changes in expression detected using data from the *Arabidopsis* or *T. salsuginea* arrays hybridized with either Col-0 or Yukon RNA. To ensure valid comparisons, all *T. salsuginea* genes were matched with *Arabidopsis* genes using BLASTX.

In fact, approximately half of the probes were detected as background signals (Additional file 15) when *T. salsuginea* RNA was hybridized to *Arabidopsis* arrays and 58% (2,553 of 4,425) of the genes differentially expressed during cold acclimation that were detected using *T. salsuginea* arrays were not identified as significantly changed using *Arabidopsis* arrays (Figure 7). A similar situation was evident when Col-0 RNA was hybridized to both arrays, where 82% (422 out of 512) of the genes that were found significantly cold-regulated using the same-species arrays showed no significant regulation in cross-species array hybridizations (Figure 7).

To facilitate data visualization and biological interpretation of array hybridization results, a *T. salsuginea* ontology/mapping file (available freely on-line at [63]) was developed by transferring the *Arabidopsis* MapMan ontology [42] to the *T. salsuginea* transcripts for use with the MapMan software [42,43].

**Figure 8:**
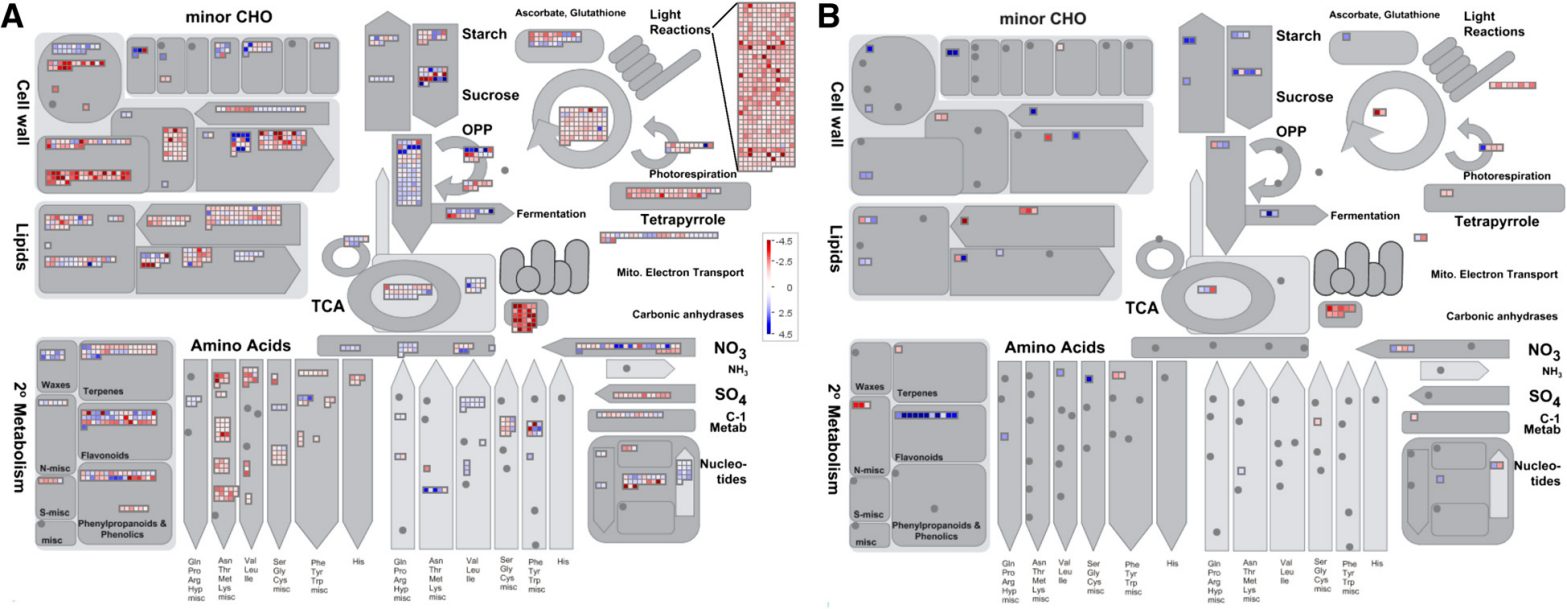
**An overview of cold responsive genes annotated in MapMan functional groups for primary and secondary metabolism.** Comparison of **(A)***T. salsuginea* and **(B)***Arabidopsis* genes encoding proteins involved in primary and secondary metabolisms. Samples derived from *T. salsuginea* Yukon were hybridized against *T. salsuginea* arrays, samples derived from *Arabidopsis* Col-0 to *Arabidopsis* arrays. Differential gene expression was determined in response to 14 days of cold acclimation at 4°C. Significance of expression changes was determined with a threshold of p < 0.05 after false discovery correction by fitting the data to a linear model. Blue indicates significantly up-regulated and red significantly down-regulated genes. Note the large difference in the number of regulated genes in the light reactions bin between the two species.

Mapping of cold-responsive genes from same-species microarrays to the MapMan ontology showed several changes in the expression of genes related to primary and secondary metabolisms, and photosynthesis (Figure 8). The proportion of cold-responsive genes in different functional groups was tested for significant deviation from the expected value for all genes in these groups represented on the arrays. MapMan functional groups that were significantly (FDR, *p* < 0.05) under- or over-represented were identified using Fisher’s exact test with false discovery rate corrected *p*-values [64]. Many functional groups were affected for either up- or down-regulated genes in *T. salsuginea* plants cold acclimated at 4°C for two weeks (Additional file 16). The functional groups that were most significantly affected when considering cold-repressed genes were photosynthesis (*p* = 1.49E-129), cell wall (*p* = 1.38E-26) and hormones (*p* = 4.45E-15). The most affected functional groups for cold-induced genes were glycolysis (*p* = 4.45E-12), stress (*p* = 6.16E-12) and biodegradation of xenobiotics (*p* = 1.58E-7). The genes in the stress bin include 26 genes classified into the sub-bin stress.abiotic.cold (*p* = 4.45E-12). These results suggest that the newly designed *T. salsuginea* array reliably detected genes or pathways that were known to be differentially affected on the transcript level in *Arabidopsis* during plant cold acclimation [18,65-69].

## Discussion

*T. salsuginea* has many features similar to *Arabidopsis* and is in addition considered an “extremophile” with a higher tolerance to various abiotic stresses and has therefore been proposed as an alternative model species to *Arabidopsis*[9]. While the genomes of *T. salsuginea* and of the related species *T. parvula* have recently been sequenced [11-13], genome annotation is still far from complete and, due to the low number of ESTs available, evidence for transcriptional activity is also scarce for most genes. In addition, the effective use of *T. salsuginea* for functional genomics investigations of mechanisms of plant abiotic stress tolerance is severely hampered by a lack of affordable tools for genome-wide gene expression studies. We therefore used next generation sequencing to obtain comprehensive information about the *T. salsuginea* transcriptome and used this information for gene annotation and the development of a dedicated microarray for global expression studies. To cover the transcriptome as fully as possible, we performed 454 pyrosequencing on ESTs generated from both a non-normalized and a normalized library synthesized from RNA samples pooled from a wide range of plant samples from different developmental stages and from plants grown under different abiotic stress conditions. Although the number of reads obtained from the normalized library was only about half of that obtained from the non-normalized library, both libraries yielded a large number of unique sequences that were not detected in the other library, indicating that sequencing of normalized and non-normalized libraries is necessary to obtain optimal coverage of a transcriptome.

A combination of 44,551 publicly available ESTs and more than 1 million new sequence reads were assembled into 42,810 contigs for the prediction of gene functions. Of these contigs, 33,147 were novel because they were not covered by publicly available *T. salsuginea* ESTs. In total, the number of publicly available *T. salsuginea* unigenes increased more than 3-fold (from 13,073 to 42,810) with the present study. The number of genes estimated from the two draft genomes of *T. salsuginea* is 29,509 [11] and 26,351 [12], while the draft genome of *T. parvula* contains 30,419 genes [13]. This suggests that the unigenes identified in this study represent a comprehensive *T. salsuginea* transcriptome. The average contig/unigene length in our assembly is 567 bp, which is shorter than the length of the predicted Shandong gene models (1,224 bp; [12]). The likely reason for this discrepancy is the fragmentation procedure necessary for the construction of normalized cDNA libraries. Nevertheless, more than 50% of our unigenes are full length (21,228 out of 42,810) based on the BLASTN alignment (E-value < 10^-10^ and query coverage > 90% in relation to Shandong gene models). In addition, 87.5% of our unigenes (37,447 out of 42,810) were mapped to Shandong gene models and 94.6% (40,480 out of 42,810) were mapped to the Shandong genomic sequence [12]. These results indicate that most of our sequence assembly is correct based on its high similarity to a closely related accession.

Many SNPs were found between our Yukon unigenes and the Shandong gene models [12] when the Yukon unigenes were aligned with the reference Shandong gene models (Table 3, Additional file 1). The significance of the putative SNPs in the context of abiotic stress tolerance or other phenotypic variation between the Yukon and Shandong accessions remains to be determined.

Based on the MapMan ontology [42,43], functional annotation of both *T. salsuginea* unigenes and *Arabidopsis* genes revealed similar proportions of genes in each functional group, with the exception of genes annotated as encoding proteins with a function in photosynthesis, which were more abundant in *T. salsuginea*, and genes encoding proteins annotated as functioning in DNA metabolism, which were less abundant. A similar observation was also made when we identified 12,272 Yukon unigenes that were likely derived from gene duplication events relative to Shandong gene models (Additional file 17). These duplicated unigenes map for most of their length (coverage > 90%) over Shandong genes and are significantly enriched for functional categories of protein synthesis, lipid metabolism, photosynthesis and abiotic/biotic stress (Table 2). The higher abundance of photosynthesis-related genes, together with the large number of such genes that were found to be regulated during cold acclimation in the microarray study, may be related to the importance of an effective regulation of photosynthesis for the abiotic stress tolerance of plants to sustain growth and avoid oxidative damage (see [70] for a review).

The percentage of genes in different functional groups in *T. salsuginea* that were predicted to be targeted by miRNAs was generally lower than the corresponding percentage in *Arabidopsis*, except for target functions related to protein metabolism and mitochondrial electron transport, where *T. salsuginea* showed much higher proportions. Whether any of the relevant miRNAs play a role in abiotic stress tolerance, however, remains to be investigated. In addition, some genes encoding LEA proteins were present in higher copy numbers of orthologous genes in the *T. salsuginea* than in the *Arabidopsis* genome. This is in agreement with the observed higher frequency of gene duplications in the *T. salsuginea*[11] and *T. parvula* genomes [13]. This may suggest that both *Thellungiella* species retained a higher fraction of duplicated genes since the last whole genome duplication event [71,72] or after tandem duplications that diverged in function (neofunctionalization; [73]) as an adaptive strategy to survive in habitats with harsher environmental stress conditions than *Arabidopsis*[74].

On the other hand, the analysis of PP2C, PTP(DSP) and MAPK(K) in *T. salsuginea, T. parvula* and *A. thaliana* indicated very similar numbers of putative genes and proteins of these families in all three species. This may indicate that the retention of duplicated genes was highly selective, a hypothesis that was also put forward in the most recent *T. salsuginea* genome sequencing study [12].

The assembled unigenes were used for the construction of a 44 K Agilent microarray that showed superior specificity for *T. salsuginea* compared to *Arabidopsis* samples, high reproducibility and the ability to detect the complex cold-acclimation induced changes in transcript abundance. In addition, it showed interesting differences in the cold regulated transcript patterns between *Arabidopsis* and *T. salsuginea*. Interestingly, a previous paper provided evidence for possible differences in the metabolic adaptation strategies of the two species [6], based on targeted biochemical measurements of compatible solutes. In the future, the availability of microarrays will allow us to investigate such differences in adaptation strategies at the functional genomics level.

The *T. salsuginea* microarray consists of 43,800 oligonucleotide probes representing 41,563 unigenes. Based on a BLASTN search of all unigenes against the *T. salsuginea* genomic contig sequences [11], 37,622 *T. salsuginea* unigenes were aligned to the published genome sequence. The remaining 5,188 unigenes showed no significant BLAST hits and filtering out unigenes that were not represented on the array still left 4,092 unigenes that had produced above-background signal intensities in the hybridization experiments performed during the pre-selection procedure. Likely reasons for this discrepancy are that approximately 10% of the genome have not been sequenced yet [11], that the corresponding transcripts are encoded by genes that consist of short exons and long introns that may result in low BLASTN hit score values, or that these unigenes have relatively high sequencing or sequence assembly errors. The latter point may have resulted in the relatively high fraction of 71% (2,942 out of 4,092) among these unigenes that were annotated as having an unknown function, while for the whole unigene set this fraction was about 50%.

To assess whether the use of a dedicated *T. salsuginea* microarray really yields data of a higher quality than cross-species hybridization on an *Arabidopsi*s array from the same manufacturer, we hybridized identical samples from either non- or cold-acclimated plants on same-species *T. salsuginea* and cross-species *Arabidopsis* arrays. In the reciprocal experiment, *Arabidopsis* samples were also hybridized to the same set of arrays. The results demonstrated the expected bimodal signal distribution [61,62]. However, only signals obtained from same-species array hybridizations showed a distribution consistent with a high proportion of specific hybridization reactions and a low proportion of non-specific hybridizations and noise. In fact, using the 25-mer oligonucleotide-based Affymetrix GeneChip ATH1, only 20% of the probes produced signals significantly above background with *T. salsuginea* samples [17]. In contrast, using longer oligonucleotide probes (70-mer) showed that approximately 80% of the probes on the *Arabidopsis* microarray [75] yielded significant signals after hybridization with *T. salsuginea* samples. However, the detection of differentially expressed *T. salsuginea* genes was limited to *Arabidopsis* homologs with an overall sequence identity of 92-95% with this type of array [2]. Obviously, the expression of *T. salsuginea*-specific unigenes could not be detected using *Arabidopsis*-based arrays and approximately 58% (2,553 out of 4,425) of the cold-responsive genes detected using the same-species array were not identified as differentially expressed from the *Arabidopsis* array, further stressing the need for a dedicated *T. salsuginea* microarray for the reliable identification of stress-regulated genes at the genomic level.

One of the challenges faced when using microarrays is to visualize the expression data in an efficient way [76]. To make the *T. salsuginea* microarray as useful as possible for the research community, we adapted the MapMan visualization [43,77] and the Robin [78] and mefisto statistical analysis tools to *T. salsuginea* by designing a mapping file for the annotated unigenes by transfer into the MapMan ontology [42]. To validate its performance, the *T. salsuginea* microarray was used to identify cold-responsive genes in comparison to *Arabidopsis*. The results were visualized using the MapMan tool. Further functional analysis was provided by statistical tests for the significant under- or over-represention of cold regulated genes in different functional groups. These results demonstrate that the newly designed *T. salsuginea* microarray reliably detects genes and pathways that are known to be differentially affected during cold acclimation in *Arabidopsis* and other plants. We are currently using the *T. salsuginea* array to investigate the cold and salt responsiveness of the *T. salsuginea* transcriptomes in the diverse set of accessions from all three Thellungiella species recently described [6].

## Conclusions

While model species such as *Arabidopsis* are valuable tools to study many important traits in plants, additional models are needed to investigate the adaptation mechanisms to more extreme environmental conditions. *T. salsuginea* is such an emerging model species and the present study provides the first comprehensive transcriptome information for this species. The large number of additional publicly available unigene sequences will greatly facilitate genome annotation. In addition, the newly designed and validated genome-wide microarray for *T. salsuginea*, which will be commercially available, will be an important tool for future functional genomics investigations of plant stress tolerance. Also, we hope that the approaches developed in this study will help in the establishment of similar genomic tools for other non-model species with interesting physiological and ecological traits.

## Methods

### Plant material and abiotic stress treatments

Seeds of the *Thellungiella salsuginea* ((Pallas) O.E. Schulz) (*Eutrema salsugineum*) accession Yukon were kindly provided by Prof. Ray A. Bressan (Purdue University, West Lafayette, IN). For transcriptome sequencing, plants were either grown in soil or hydroponically under non-sterile conditions in a greenhouse at 16-h day length with light supplementation to reach at least 200 μE m^-2^ s^-1^ and a temperature of 20°C during the day, 18°C during the night. The nutrient solution for hydroponically-grown plants contained 1 mM MgSO_4,_ 1 mM KH_2_PO_4_, 1 mM NH_4_NO_3_, 0.5 mM CaCl_2_, 0.1 mM FeNA-EDTA, 25 mM NaCl, 0.1 mM H_3_BO_3_, 1.5 μM CuSO_4_, 50 μm KCl, 10 μm MnSO_4_, 0.075 μM Na_2_MoO_4_, 0.1 mM Na_2_O_3_Si, and 2 μM ZnSO_4_. Six-week-old soil-grown and five-week-old hydroponically-grown plants were treated for 24 h or 2 weeks at 4°C in a growth cabinet at 16 h day length with 90 μE m^-2^ s^-1^, or stressed with 200 mM NaCl solution by irrigation or supplementation of the nutrient solution. For drought treatment, water was withheld from soil-grown plants for 5 days (mild drought with slight curling of leaves) or 10 days (severe drought with strong curling of leaves). Shoots and roots were collected from soil- and hydroponically-grown plants, except for soil-grown roots after stress treatments. In addition, rosette leaves, cauline leaves, opened flowers and siliques were collected from control plants grown in soil or hydroponically. For all samples, material from 5 to 10 individual plants was pooled. All samples were immediately frozen in liquid nitrogen and stored at −80°C.

For the microarray experiments, *T. salsuginea* seeds were sown in soil and exposed to 4°C under the conditions described above for one week to promote germination. Seedlings were transferred to a greenhouse as above for 8 weeks (nonacclimated plants). For cold acclimation, plants were transferred to a 4°C growth cabinet under the conditions described above for an additional 14 days [6]. *Arabidopsis* plants were grown and acclimated under identical conditions [79,80], but were grown under nonacclimating conditions for 6 weeks to reach the same developmental state [6].

### RNA isolation, library construction and sequencing

Total RNA was isolated and on-column DNA digestion was performed with the RNeasy Plant mini kit (Qiagen, Hilden, Germany) according to the manufacturer’s protocol. Equal amounts of RNA from 23 individual samples obtained as described above were pooled and cDNA was synthesized with the Mint-Universal kit (Evrogen, Moscow, Russia). Half of the cDNA was normalized to equalize the abundance of transcripts by using the duplex-specific nuclease normalization approach [81] with the TRIMMER-DIRECT cDNA normalization kit (Evrogen) according to the manufacturer’s protocol. The cDNA in both libraries was size-fractionated using Chroma Spin-1000 columns (Clontech, Mountainview, CA). 454 libraries were constructed using custom-made adapters with MIDs (Multiplex Identifiers) following the Roche Technical Bulletin TCB 09004, introducing *Sfi*I-sites for creating directed sequencing libraries. The 454 libraries were immobilized on beads and clonally amplified using the GS FLX Titanium LV emPCR Kit (Lib-L) (Roche, Basel, Switzerland). The libraries were then sequenced using the GS FLX Titanium Sequencing Kit XLR70 (Roche) and GS FLX Titanium PicoTiterPlate Kit (Roche) according to the manufacturer’s protocols.

### Transcriptome assembly and annotation

Adapter and polyA sequences were removed from the data with the SFF Tools of the Genome Sequencer FLX System software package 2.3 (Roche) and the cross_match (http://www.phrap.org) algorithm. The clipped reads were pre-checked for contamination by non-plant sequences using BLAST [82] searches in the NCBI database of non-redundant sequences (nr; updated at 12-02-2010) with an E-value threshold of 10^-10^. All reads matching as best hit a sequence originating from an organism outside the Streptophyta phylum were considered “contaminant”. These reads were a minor part of the population and were thus included during the assembly process, but contigs including them were marked as “contaminant contigs”. The assembly was conducted using the MIRA assembler program version 3.1.15 [36] with default settings and the 454 reads from the normalized, the non-normalized and the combined libraries. To improve the accuracy of sequence assembly, we downloaded the 44,551 publicly available *T. halophila* and *T. salsuginea* ESTs from GenBank, NCBI (as of April 19, 2010) and included them in all assemblies. Reads not alignining to any other read (singletons) were not included in the final contig population. Average contig coverage was calculated as the mean number of reads per base per contig. The N50 was calculated as the contig length above which 50% of the sequence information is contained, i.e. 50% of the nucleotides in the output are contained in contigs of length N50 or longer.

All contigs were checked for presence of open reading frames (ORFs) as previously described [83]. To assess the completeness of the transcriptome assemblies and the degree of overlap between *T. salsuginea* and *Arabidopsis*, we used BLASTX to align the contig sequences to the 27,739 *Arabidopsis* Information Resource (TAIR9; [84]) peptide sequences [85]. We calculated the percentage of *Arabidopsis* proteins matching *T. salsuginea* contigs with a loose threshold to account for interspecies variation (E-value < 10^-3^). All following steps were conducted on the assembly based on the combined library only. Since the population of plants used for RNA extraction was not homozygous, SNPs generated several nearly identical contigs. We aligned each contig to all contigs in the population via BLAST, to identify clusters of contigs matching each other with a sequence coverage and identity higher than 99%. A multiple alignment was produced for each cluster using MUSCLE [86]. Consensus sequences for each cluster were extracted from the multiple alignments using the *consambig* tool from the EMBOSS suite [87]. Where disagreeing base pairs were found, the resulting cluster sequence was dubbed using the International Union of Pure and Applied Chemistry (IUPAC) code for nucleotide ambiguity.

Functional classification of the putative transcripts was performed using the Mercator pipeline [41]. Mercator aligns all sequences against five different databases: TAIR9 proteins [85], SwissProt/Uniprot plant proteins (PPAP) [88], Conserved Domain Database (CDD) [89], Clusters of Orthologous Groups (COG) [90], and InterProScan [91] and subsequently computes preliminary MapMan bin [43] codes/ontology based on manually curated reference classifications using a majority vote scheme. The programs used to perform the searches were RPSBLAST [92] for CDD and COG and BLASTX [82] for TAIR9 and PPAP. Hits with bit scores lower than 50 were ignored as not significantly similar. The sequencing procedure does not guarantee that the orientation of the original mRNAs is kept. Hence any of the 42,810 transcripts can be either 5″-3′ or 3′-5′ oriented. To unify the orientation, we used the protein models present in the NCBI nr database and, where available, we used the best BLAST hit (E-value < 10^-3^) to define the orientation of the transcript.

To estimate the number of *T. salsuginea* unigenes that were orthologs of *Arabidopsis* or *T. parvula* genes, we used BLASTX to search the unigenes with a stringent threshold (E-value < 10^-10^) against *Arabdopsis* TAIR10 database or coding sequence of *T. parvula* genome assembly version 2 [13]. To estimate the number of *T. salsuginea* unigenes/transcripts that were derived from the *T. salsuginea* genome, we used BLASTN to search the unigenes with a stringent threshold (E-value < 10^-50^) against the *T. salsuginea* genome assembly [11]. In addition, we took advantage of the recently published gene models of the Shandong accession of *T. salsuginea* (*Eutrema salsugineum*) [12] by comparing them to the unigenes from the Yukon accession used in our study, using BLASTN with an E-value < 10^-10^. We estimated the full length genes of Yukon by aligning the unigene sequences with Shandong gene models using BLASTN (E-value < 10^-10^) and > 90% coverage of the aligned query region with the nucleotide sequence length of the published gene models (query coverage). An estimate of single nucleotide variation between Shandong gene models and Yukon unigenes was obtained using VarScan version 2.3.5 with default parameters [37] with Shandong gene models [12] as reference sequences. Ambiguous single nucleotide variations (more than one nucleotide variation at a given nucleotide position) were filtered out.

Orthologs were identified to determine duplicated genes among Yukon unigenes relative to Shandong gene models based on BLASTN analysis (E-value < 10^-10^) and aligned query coverage > 90% compared to Shandong gene models.

All *T. salsuginea* contigs were searched by BLASTN against the complete set of miRNA precursor transcripts from the plant miRNA database [93] with an E-value < 10^-10^. The functions of the predicted miRNAs were inferred from the target transcripts that were extracted based on the tables provided as search results [48].These inferred functions were classified based on MapMan ontology. Bincodes were truncated at the primary level of the hierarchy. We aslo performed a similar miRNA prediction of *Arabidopsis* TAIR9 transcripts and classified their functions based on MapMan ontology with identical search parameter settings.

PP2C/DSP/PTP/MAPK or MAPKK protein sequences derived from the *A. thaliana*, *T. parvula* and *T. salsuginea* genomes (http://www.arabidopsis.org/, [11,13]) were compared to *T. salsuginea* unigenes using BLASTN (e < 10^-20^). BLASTX (e < 10^-60^) scans were performed using putative *T. salsuginea* protein models to verify BLASTN hits [94]. Unigenes were translated according to BLASTX results. Individual *T. salsuginea* putative MAPKK, MAPK, PP2C, DSP and PTP proteins were designated according to the corresponding orthologous protein annotations in *Arabidopsis*. Multiple alignments of *Arabidopsis* and *T. salsuginea* putative PP2Cs, PTPs, DSPs, MAPKKs and MAPKs were performed using MUSCLE [86,95] and were edited manually afterwards. Unrooted phylogenetic trees were computed with UGENE [96] using a JTT model with standard parameters (gamma distribution and a coefficient of variation of substitution rates among sites of 0.5).

Genes encoding putative LEA proteins of *T. salsuginea* were identified based on reciprocal BLASTX (E < 10^-10^) searches of the 51 *Arabidopsis* LEA genes [58] against all *T. salsuginea* unigenes. All resulting LEA proteins were aligned using ClustalW and an unrooted dendogram was drawn [97]. GRAVY (grand average of hydropathy) values were determined using protparam [98]. Subcellular localization was predicted from protein sequence analysis using TargetP [99].

### Microarray design and hybridization

We designed a *T. salsuginea* microarray based on the custom gene expression 4 × 44 K platform from Agilent, consisting of four arrays per slide with 45,220 features, 43,803 of which are user-defined 60-mer oligonucleotide probes. Probes and microarrays were designed by imaGenes GmbH (Berlin, Germany). Briefly, 415,000 oligonucleotide probes were designed from the 42,810 contig sequences and printed as 2 × 400 K arrays. The same pooled RNA that was used for the sequencing libraries was used for the synthesis of Cy3-labeled cRNA that was hybridized to one of these arrays. Genomic DNA was extracted from rosette leaves of 6-week-old *T. salsuginea* plants using the DNeasy mini kit (Qiagen). Fragmented and labeled genomic DNA was hybridized to the other 400 K array. The 43,800 best-performing probes were selected and submitted to Agilent eArray platform to fabricate 4 × 44 K arrays. The arrays are commercially available from Agilent [60] with the design number 031554.

### Microarray data analysis

The feature extraction files were analyzed using the Limma bioconductor package implemented in the Robin/RobiNA tool [78,100]. Background signals were subtracted and values were quantile-normalized before a linear model was fitted for statistical testing of differential expression. Transcripts showing a log_2_ fold change ≥ 1 or ≤ −1 with a *p*-value below 0.05 after false discovery rate correction [64] in the contrast between cold acclimated and nonacclimated samples were accepted as significantly changed. Non-acclimated replicate 1 of Col-0 hybridized to the *Arabidopsis* array was an outlier and was excluded from the analysis of differential gene expression. Otherwise, all RNA samples were included in all analyses. Statistical comparisons of functional groups of cold-responsive genes were made against all genes in a particular group represented on the array using Fisher’s exact test implemented in the mefisto software [101]. Significantly regulated genes were considered as significantly over- or under-represented in a MapMan functional group at *p* < 0.05 after false discovery rate correction [64]. Microsoft Excel 2010 and the R software [102] were used to perform general analyses.

### Availability of supporting data

The 454 reads for *Thellungiella salsuginea* reported here have been submitted to NCBI sequence read archive (SRA, http://www.ncbi.nlm.nih.gov/sra) under the accession numbers SRX197603 and SRX197604. Microarray hybridization results are available at GEO (http://www.ncbi.nlm.nih.gov/geo) under the accession number GSE42156.

## Competing interests

The authors declare that they have no competing interests.

## Authors’ contributions

YPL and SK performed the experiments, YPL, RR and DKH designed the project, YPL, FMG, ML, KK, BU and IM performed data analysis and provided bioinformatic tools, YPL and DKH drafted the manuscript, all authors participated in manuscript writing. All authors read and approved the final manuscript.

## Supplementary Material

Additional file 1 **A list of non-ambiguous single nucleotide variations identified between Shandong gene models [**[12]**] and Yukon unigenes using VarScan [**[37]**].** Click here for file

Additional file 2 ***Arabidopsis *** **MAPK and MAPKK genes and the corresponding *T. salsuginea *** **contigs.** The indicated groups refer to the *Arabidopsis* MAPK and MAPKK protein nomenclature [103]. Click here for file

Additional file 3 ***Arabidopsis *** **PP2C genes and the corresponding *T. salsuginea *** **contigs.** The indicated clusters refer to the *Arabidopsis* PP2C protein nomenclature [52] and the phylogenetic tree shown in Additional file 5. Click here for file

Additional file 4 ***Arabidopsis *** **PTP and DSP genes and the corresponding *T. salsuginea *** **contigs.** Click here for file

Additional file 5 **Unrooted phylogenetic tree of all *Arabidopsis *** **and *T. salsuginea *** **PP2C proteins shown in Additional file****3****.** Click here for file

Additional file 6 **Unrooted phylogenetic tree of all *Arabidopsis *** **and *T. salsuginea *** **PTP and DSP proteins shown in Additional file****4****.** Click here for file

Additional file 7 **Unrooted phylogenetic tree of all *Arabidopsis *** **and *T. salsuginea *** **MAPK and MAPKK proteins shown in Additional file****2****.** The indicated groups refer to the *Arabidopsis* MAPK and MAPKK protein nomenclature [103]. Click here for file

Additional file 8 **List of all genes encoding MAPK, MAPKK, PP2C, PTP and DSP proteins in *Arabidospis *** **and of all putative genes encoding orthologous proteins in *Thellungiella salsuginea.*** The table contains the gene identifiers, the number of encoded amino acids for each gene and the derived protein sequences. Click here for file

Additional file 9 **List of all genes encoding LEA proteins in *Arabidospis *** **and of all putative genes encoding orthologous LEA proteins in *T. salsuginea.*** The table contains the gene identifiers, the number of encoded amino acids for each gene and the derived protein sequences. Click here for file

Additional file 10 **Unrooted dendogram of all *Arabidopsis *** **and *T. salsuginea *** **LEA proteins.** The sequence numbers and their corresponding protein sequences are listed in Additional file 9. Click here for file

Additional file 11 **Assignment of putative *T. salsuginea *** **LEA proteins to different protein family domains based on multiple sequence alignment and phylogeny analyses of putative *T. salsuginea *** **and known *Arabidopsis *** **LEA proteins.** Click here for file

Additional file 12 **Characteristics of *T. salsuginea *** **LEA proteins.** Click here for file

Additional file 13 Reproducibility of signal intensities of all hybridized probes among biological replicates of non-acclimated (NA) and cold acclimated leaf samples (ACC). Click here for file

Additional file 14 **Correlation of fold change of different probes designed from the same contigs.** Log_2_-transformed fold change of mean signal intensities for 2,237 probe pairs from the same contigs for the three biological replicates. Click here for file

Additional file 15 **Number of probes identified from the reciprocal hybridization of RNA samples derived from non-acclimated or cold acclimated leaves to *Arabidopsis *** **and *T. salsuginea *** **arrays.** Click here for file

Additional file 16 Statistical analysis showing over- and under-representation of MapMan functional groups among significantly cold regulated genes. Click here for file

Additional file 17 **List of putative duplicated Yukon unigenes with respect to Shandong gene models.** The annotations provided are derived from Mercator and Mapman software (Yukon unigenes) and from the Phytozome annotation (Shandong gene models). Click here for file
