# Supplementary material for: Transcriptome sequencing and microarray design for functional genomics in the extremophile Arabidopsis relative Thellungiella salsuginea (Eutrema salsugineum)
Source: BMC Genomics. 2013 Nov 14;14:793. doi: 10.1186/1471-2164-14-793 (PMC3832907; doi:10.1186/1471-2164-14-793)
Supplement: Additional file 2 — Arabidopsis MAPK and MAPKK genes and the corresponding T. salsuginea contigs. The indicated groups refer to the Arabidopsis MAPK and MAPKK protein nomenclature [103]. [file 1471-2164-14-793-S2.pdf]

Additional file 2.

AGI	MAPK	Contigs	
AT3G45640	ATMPK3	thellun_all_c7986; thellun_all_c29917	A
AT2G43790	ATMPK6	thellun_all_c38895; thellun_all_c17154	
AT4G01370	ATMPK4	thellun_all_c8037; thellun_all_c7456	B
AT4G11330	ATMPK5	thellun_all_c37344	
AT2G46070	ATMPK12	thellun_all_c28023	
AT1G10210	ATMPK1	thellun_all_rep_c12627	C
AT1G59580	ATMPK2	thellun_all_c16994; thellun_all_c43504	
AT2G18170	ATMPK7	thellun_all_rep_c16551; thellun_all_rep_c39120	
AT1G18150	ATMPK8	thellun_all_rep_c15999; thellun_all_c29966	D
AT2G01450	ATMPK17	thellun_all_c22266; thellun_all_c5989; thellun_all_c22856	
AT1G53510	ATMPK18	thellun_all_c12164; thellun_all_c25005; thellun_all_c28956	
AT3G14720	ATMPK19	thellun_all_c34090; thellun_all_c33231; thellun_all_rep_c27519	
AT2G42880	ATMPK20	thellun_all_c7287	

AGI	MAPKK	Contigs	
AT4G26070	ATMKK1	thellun_all_c26223	A
AT4G29810	ATMKK2	thellun_all_c14205; thellun_all_c31759	
AT5G40440	ATMKK3	thellun_all_c19964	B
AT1G51660	ATMKK4	thellun_all_c13244; thellun_all_c21648	C
AT3G21220	ATMKK5	thellun_all_c12458	
AT1G73500	ATMKK9	thellun_all_rep_c5758	D

.
