# Supplementary material for: Transcriptome sequencing and microarray design for functional genomics in the extremophile Arabidopsis relative Thellungiella salsuginea (Eutrema salsugineum)
Source: BMC Genomics. 2013 Nov 14;14:793. doi: 10.1186/1471-2164-14-793 (PMC3832907; doi:10.1186/1471-2164-14-793)
Supplement: Additional file 3 — Arabidopsis PP2C genes and the corresponding T. salsuginea contigs. The indicated clusters refer to the Arabidopsis PP2C protein nomenclature [52] and the phylogenetic tree shown in Additional file 5. [file 1471-2164-14-793-S3.pdf]

Additional file 3

PP2C	Contigs	
AT1G17550	thellun_all_c15240; thellun_all_c8281	A
AT1G72770	thellun_all_c42497; thellun_all_c35551; thellun_all_c35976; thellun_all_c12890	A
AT4G26080	thellun_all_c5358; thellun_all_rep_c30083; thellun_all_rep_c46466; thellun_all_rep_c46546; thellun_all_rep_c47056; thellun_all_rep_c2155; thellun_all_c10510	A
AT5G57050	thellun_all_c7659	A
AT5G59220	thellun_all_c10441; thellun_all_c39203	A
AT1G67820	thellun_all_c29263; thellun_all_c11178	B
AT2G30020	thellun_all_rep_c21558	B
AT2G28890	thellun_all_c12283; thellun_all_c43105	C
AT2G46920	thellun_all_c32522	C
AT3G16560	thellun_all_c28902; thellun_all_rep_c29744; thellun_all_c20473; thellun_all_c40706; thellun_all_c8999	C
AT5G02400	thellun_all_c33736	C
AT3G12620	thellun_all_c15921; thellun_all_rep_c8878; thellun_all_c15921	D
AT3G17090	thellun_all_c17963; thellun_all_c20283; thellun_all_c31363	D
AT4G33920	thellun_all_c32155; thellun_all_c17437	D
AT5G02760	thellun_all_rep_c5904; thellun_all_rep_c16851	D
AT5G06750	thellun_all_c19251	D
AT5G66080	thellun_all_c44914	D
AT1G03590	thellun_all_c40460	E
AT1G16220	thellun_all_c18413; thellun_all_rep_c12058	E
AT1G79630	thellun_all_c44459; thellun_all_c32998; thellun_all_c28268	E
AT2G20050	thellun_all_c17905; thellun_all_c25830	E
AT3G02750	thellun_all_rep_c12905	E
AT3G05640	thellun_all_c24920	E
AT3G06270	thellun_all_c8831; thellun_all_c43850	E
AT3G11410	thellun_all_rep_c22578; thellun_all_rep_c5943	E
AT3G16800	thellun_all_c29733; thellun_all_rep_c6578; thellun_all_c17029	E
AT4G32950	thellun_all_c38543; thellun_all_c22535; thellun_all_c9001; thellun_all_c37836	E
AT5G26010	thellun_all_c15738	E
AT5G27930	thellun_all_c24807; thellun_all_c8153; thellun_all_c24920	E
AT5G36250	thellun_all_c38545	E
AT4G03415	thellun_all_c30555	E
AT1G22280	thellun_all_rep_c4715	F
AT1G34750	thellun_all_c28124; thellun_all_c10309; thellun_all_c13443	F
AT1G43900	thellun_all_c7285	F
AT1G78200	thellun_all_c35162; thellun_all_c41399	F
AT2G20630	thellun_all_rep_c4759; thellun_all_rep_c30416	F
AT3G15260	thellun_all_c15439; thellun_all_c23419; thellun_all_c6989	F
AT3G51370	thellun_all_rep_c6753; thellun_all_rep_c4470; thellun_all_c44914	F
AT4G28400	thellun_all_rep_c11914; thellun_all_c18352	F
AT4G31750	thellun_all_c13247; thellun_all_c30116; thellun_all_c24675; thellun_all_c19359	F
AT5G10740	thellun_all_rep_c16595	F
AT5G53140	thellun_all_c6492	F
AT1G48040	thellun_all_rep_c21154	G
AT2G25620	thellun_all_c14749	G
AT2G33700	thellun_all_c25164; thellun_all_c15172	G
AT3G51470	thellun_all_c19337	G
AT3G62260	thellun_all_c23303; thellun_all_c13434	G

PP2C	Contigs	
AT2G25070	thellun_all_rep_c6528; thellun_all_c9380	I
AT4G31860	thellun_all_c10464; thellun_all_c19719	
AT3G63340	thellun_all_c18912	J
AT5G66720	thellun_all_c8419	K
AT2G30170	thellun_all_rep_c7048; thellun_all_c28521	
AT4G16580	thellun_all_c25834; thellun_all_c14136; thellun_all_c35046	
AT2G40860	thellun_all_c18071; thellun_all_c23308	
AT4G27800	thellun_all_rep_c6391; thellun_all_rep_c4376	
AT1G18030	thellun_all_c37154	
AT4G33500	thellun_all_rep_c15908; thellun_all_c20279	
