# Supplementary material for: Transcriptome sequencing and microarray design for functional genomics in the extremophile Arabidopsis relative Thellungiella salsuginea (Eutrema salsugineum)
Source: BMC Genomics. 2013 Nov 14;14:793. doi: 10.1186/1471-2164-14-793 (PMC3832907; doi:10.1186/1471-2164-14-793)
Supplement: Additional file 4 — Arabidopsis PTP and DSP genes and the corresponding T. salsuginea contigs. [file 1471-2164-14-793-S4.pdf]

Additional file 4

PTP	Contigs
AT1G71860	thellun_all_c7717
**DSP**	**Contigs**
AT3G09100	thellun_all_c16852
AT3G19420	thellun_all_c27082; thellun_all_c7957; thellun_all_c8321
AT4G03960	thellun_all_c17019
AT3G62010	thellun_all_c27894
AT1G05000	thellun_all_rep_c5856
AT2G04550	thellun_all_rep_c40535; thellun_all_rep_c5958
AT2G35680	thellun_all_c12344; thellun_all_c7887; thellun_all_c8558
AT3G02800	thellun_all_c13438
AT3G06110	thellun_all_rep_c1481
AT3G23610	thellun_all_c14961
AT5G16480	thellun_all_c36563
AT5G23720	thellun_all_c20615; thellun_all_c33643
AT5G56610	thellun_all_c6967
AT3G50110	thellun_all_c10116; thellun_all_c11894
AT5G58160	thellun_all_c41093
AT3G52180	thellun_all_rep_c3791
AT3G10940	thellun_all_rep_c4495
AT3G01510	thellun_all_c19238; thellun_all_c37920
