# Supplementary figures and images for: Transcriptome sequencing and microarray design for functional genomics in the extremophile Arabidopsis relative Thellungiella salsuginea (Eutrema salsugineum)
Source: BMC Genomics. 2013 Nov 14;14:793. doi: 10.1186/1471-2164-14-793 (PMC3832907; doi:10.1186/1471-2164-14-793)

**Additional file 6**

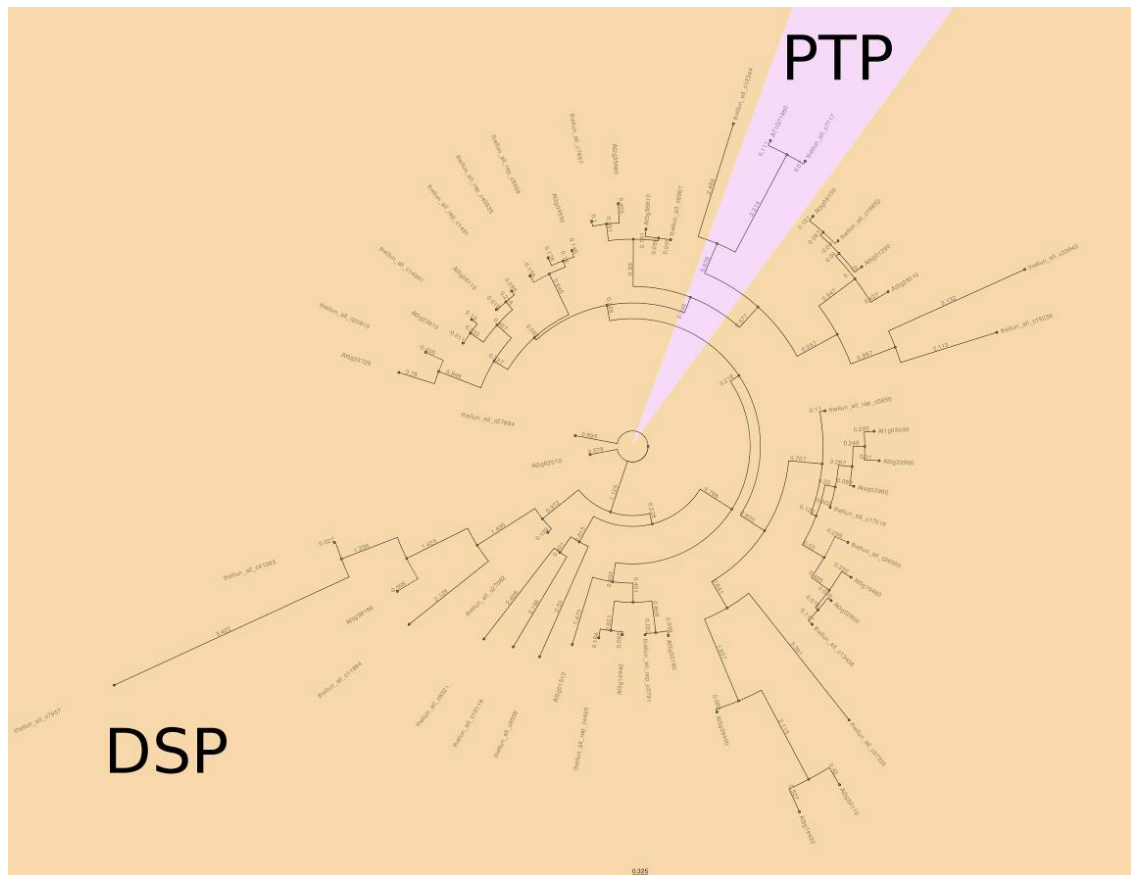

Supplement: Additional file 6 — Unrooted phylogenetic tree of all Arabidopsis and T. salsuginea PTP and DSP proteins shown in Additional file4. [file 1471-2164-14-793-S6.pdf]

Additional file 7

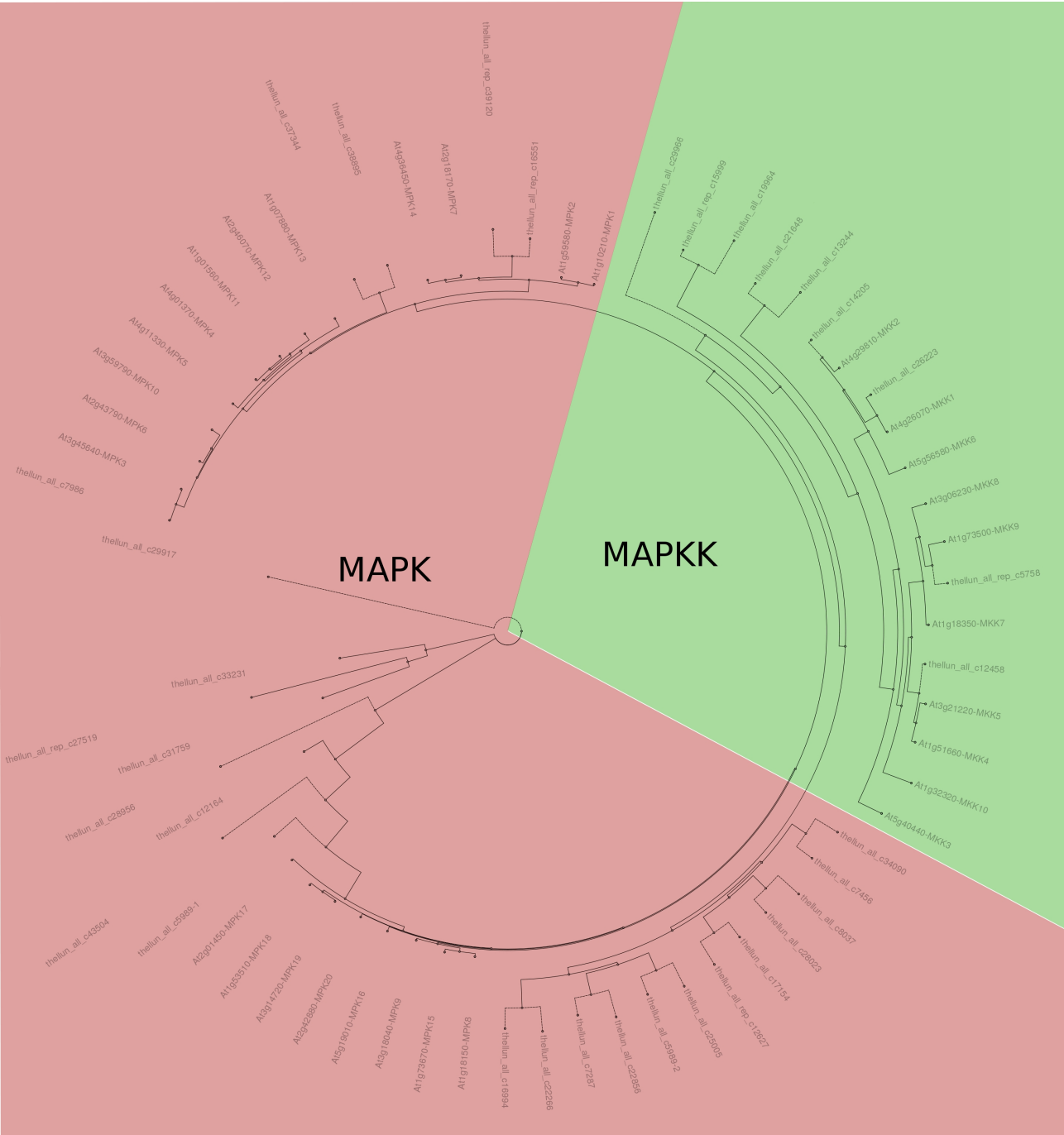

Supplement: Additional file 7 — Unrooted phylogenetic tree of all Arabidopsis and T. salsuginea MAPK and MAPKK proteins shown in Additional file2. The indicated groups refer to the Arabidopsis MAPK and MAPKK protein nomenclature [103]. [file 1471-2164-14-793-S7.pdf]

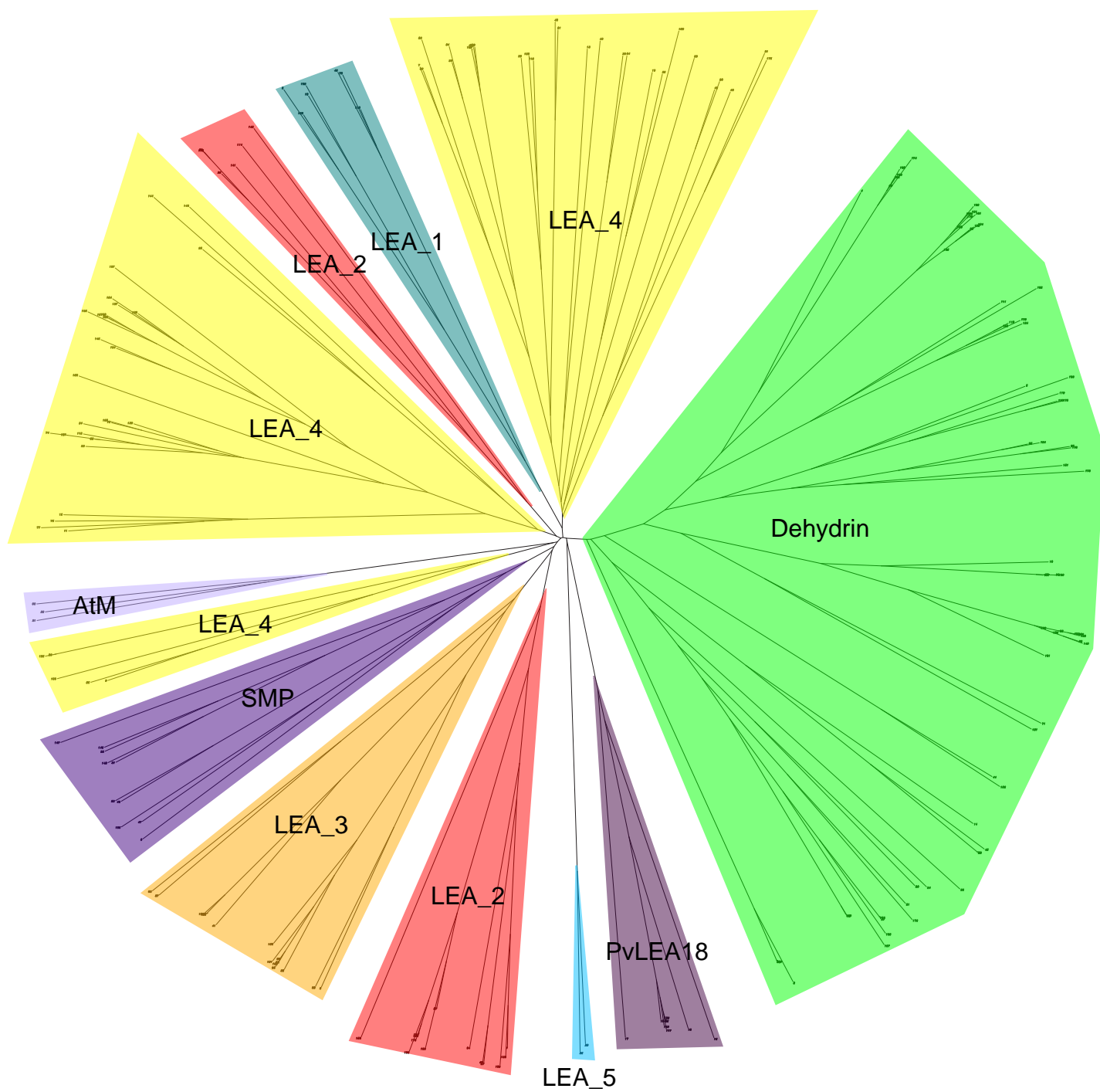

Supplement: Additional file 10 — Unrooted dendogram of all Arabidopsis and T. salsuginea LEA proteins. The sequence numbers and their corresponding protein sequences are listed in Additional file 9. [file 1471-2164-14-793-S10.pdf]

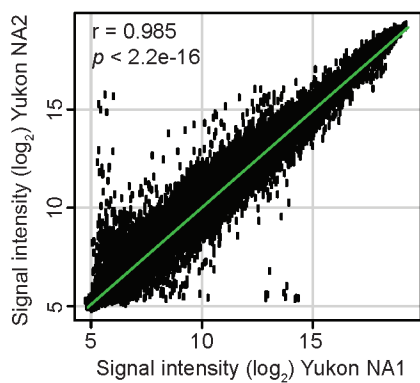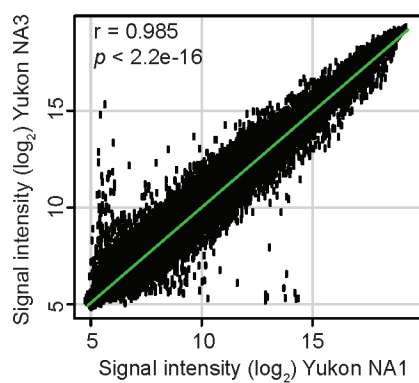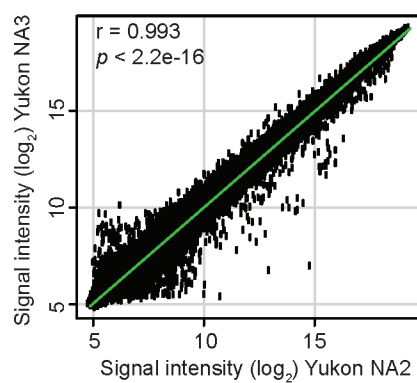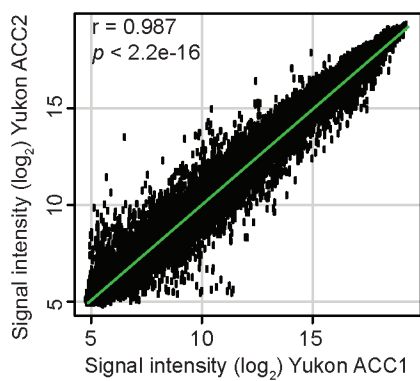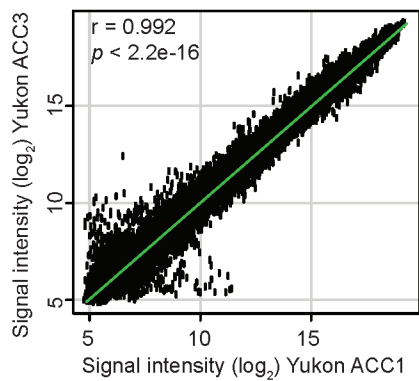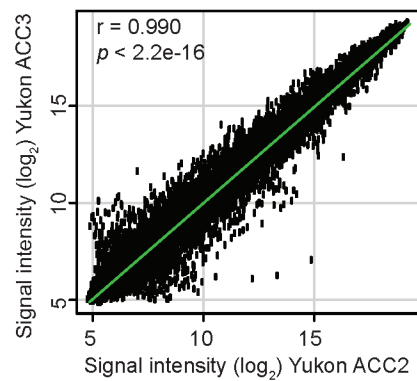

Supplement: Additional file 13 — Reproducibility of signal intensities of all hybridized probes among biological replicates of non-acclimated (NA) and cold acclimated leaf samples (ACC). [file 1471-2164-14-793-S13.pdf]

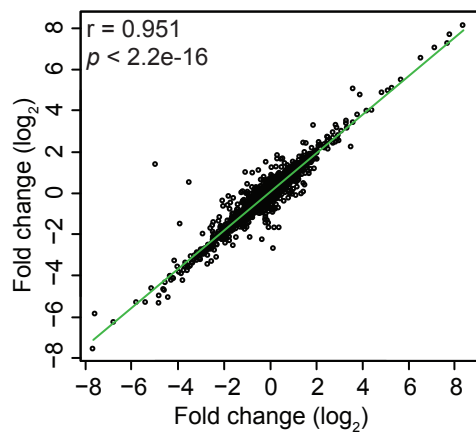

Supplement: Additional file 14 — Correlation of fold change of different probes designed from the same contigs. Log2-transformed fold change of mean signal intensities for 2,237 probe pairs from the same contigs for the three biological replicates. [file 1471-2164-14-793-S14.pdf]
