# Supplementary material for: Transcriptome sequencing and microarray design for functional genomics in the extremophile Arabidopsis relative Thellungiella salsuginea (Eutrema salsugineum)
Source: BMC Genomics. 2013 Nov 14;14:793. doi: 10.1186/1471-2164-14-793 (PMC3832907; doi:10.1186/1471-2164-14-793)
Supplement: Additional file 11 — Assignment of putative T. salsuginea LEA proteins to different protein family domains based on multiple sequence alignment and phylogeny analyses of putative T. salsuginea and known Arabidopsis LEA proteins. [file 1471-2164-14-793-S11.pdf]

Additional file 11.

Group	Ortholog of LEA proteins		Remark
	Arabidopsis	*Thellungiella*	
LEA_1	6	**133**	complete cds: 108
	18	**108**	
	46	**72**, 110	
LEA_2	1	**94**, 106, 122, 153	105 and 106 are different translated frames of thellun_all_rep_c15208; 122 and 123 are different translated frames of thellun_all_rep_c20993; 153 and 154 are different translated frames of thellun_all_rep_c28021
		**123**, 87, 105, 115, 154	
		**196**	
	26	**63**, 113	113 and 114 are different translated frames of thellun_all_rep_c17285; 141 and 142 are different translated frames of thellun_all_rep_c26673
		**142**, 114, 141	
	27	**60**	complete cds: 60
LEA_3	2	**58**	
	37	**62**	
	38	**52**, 65, 88, 132, 155, 191	complete cds: 52
	41	**54**, 100, 121, 156	complete cds: 54
LEA_4	7	**149**, 66, 67, 185, 189	complete cds: 149; 66 and 67 are different translated frames of thellun_all_rep_c4181
	9	**85**	109 matched to AT22600((no lea); 85 and 86 are different translated frames of thellun_all_c8083
		**125**, 86	
	11	**77**	
	12	**75**	
	13		
	19		
	23	**55**, 74, 112, 187	complete cds: 55, 198; 163 and 164 are different translated frames of thellun_all_rep_c34113
		**145**, 134, 135, 158, 197	
		**157**, 159	
		**198**, 137, 163, 164	
	24	**168**, 73, 139	
	25	**144**, 143	143 and 144 are different translated frames of thellun_all_c26853
	28		102 matched to AT5G38760, 116 matched to AT5G53820. Both AT5G38760 and AT5G53820 were not annotated as LEA (Hundertmark and Hincha, 2008)
	29	**84**, 83	83 and 84 are different translated frames of thellun_all_rep_7426
	30	**64**	complete cds: 64
	36	**160**	
	39		
	40	**81**	complete cds: 81
	42	**90**, 91	90 and 91 are different translated frames of thellun_all_c8752
	43		
	48	**175**	

LEA_5	20		
	35		
AtM	21		
	22	**93**	
LEA_6	15		
	16	**177**, 56, 82, 169, 184, 186	complete cds: 177
	17		
SMP	3		146, 147, 148 are translated frames of thellun_all_c27216
	31	**148**, 147	
	32	**146**	
	47	**165**	
	49		
	50		
dehydrin	4	**59**, 120, 138, 162, 174, 179, 192	161 and 162 are different translated frames of thellun_all_rep_c32321; 178 and 179 are different translated frames of thellun_all_rep_c41511
		**126**, 61, 126, 150, 161, 166, 178	
		**195**, 99, 104, 190	
	5	**95**, 97, 151, 173, 176, 194	192 and 193 are different translated frames of cluster216; 194 and 195 are different translated frames of cluster331
		**124**, 111, 118, 131,152	
		**130**, 119, 170, 193	
	8	**78**, 53, 107, 127	
	10	**98**, 70, 117, 180	complete cds: 199. Sequence 71 is translated frame +1 of thellun_all_rep_c5017 and clustered close to sequence 10. It is highly similar to mitochondrial lipoamide dehydrogenase 1 (AT1G48030) and likely not a LEA ortholog, herby not represented as ortholog of #10 in the Table.
		**181**	
		**199**, 68, 69, 76, 92, 101, 128, 140, 171, 182, 188	
	14	**89**	
	33		
	34	**80**	
	44	**103**	
	45		
	51	**57**, 79, 129, 183	complete cds: 57
		136	
		167	
		172	

Putative Thellungiella LEA proteins were identified by reciprocal BLASTX ($E < 10^{-10}$) searches of all *T. salsuginea* unigenes against the 51 *Arabidopsis* LEA proteins and resulted in 148 protein sequences as shown in Supplemental Table S2. Empty cells indicate no orthologous protein was found for *T. salsuginea* in the sequence assembly. Cds, coding sequence.
