# Supplementary material for: Transcriptome sequencing and microarray design for functional genomics in the extremophile Arabidopsis relative Thellungiella salsuginea (Eutrema salsugineum)
Source: BMC Genomics. 2013 Nov 14;14:793. doi: 10.1186/1471-2164-14-793 (PMC3832907; doi:10.1186/1471-2164-14-793)
Supplement: Additional file 12 — Characteristics of T. salsuginea LEA proteins. [file 1471-2164-14-793-S12.pdf]

Additional file 12. Characteristics of the *T. salsuginea LEA proteins*.

#[1]	AGI code	group[2]	GRAVY[2]	Localization[2]	Thellungiella orthologous[3]	#[1]	GRAVY[4]	Localization[4]
**1**	At1g01470	LEA_2	0.056	other	thellun_all_rep_c9292_3	94	0.648	other
					thellun_all_rep_c20993_3	123	-0.191	other
					cluster349_3	196	-0.022	other
**2**	At1g02820	LEA_3	-0.491	Chloroplast	thellun_all_rep_c1937_3	58	-0.437	Mitochondrion
**3**	At1g03120	SMP	-0.564	other				
**4**	At1g20440	dehydrin	-1.257	other	thellun_all_rep_c2030_1	59	-1.338	other
					thellun_all_rep_c21860_1	126	-1.176	other
					cluster331_2	195	-1.484	other
**5**	At1g20450	dehydrin	-1.34	other	thellun_all_rep_c11602_3	95	-1.169	other
					thellun_all_rep_c21096_3	124	-1.683	other
					thellun_all_rep_c23744_3	130	-1.433	other
**6**	At1g32560	LEA_1	-1.042	other	thellun_all_c24961_1	133	-0.997	other
**7**	At1g52690	LEA_4	-1.317	other	thellun_all_rep_c27290_2	149	-1.487	other
**8**	At1g54410	dehydrin	-1.868	other	thellun_all_rep_c6337_2	78	-1.254	other
**9**	At1g72100	LEA_4	-0.46	secreted	thellun_all_c8083_2	85	-0.424	other
					thellun_all_c21843_1	125	-0.495	secreted
**10**	At1g76180	dehydrin	-1.265	other	thellun_all_rep_c13061_2	98	-0.979	other
					thellun_all_rep_c45204_1	181	-0.662	secreted
					cluster597_1	**199**	-1.249	other
**11**	At2g03740	LEA_4	-0.703	Chloroplast	thellun_all_rep_c6079_3	77	-0.391	Chloroplast
**12**	At2g03850	LEA_4	-0.496	Chloroplast	thellun_all_rep_c5402_1	75	-0.686	Chloroplast
**13**	At2g18340	LEA_4	-0.93	secreted				
**14**	At2g21490	dehydrin	-1.032	other	thellun_all_rep_c8639_3	89	-0.733	other
**15**	At2g23110	PvLEA18	-1.059	other				
**16**	At2g23120	PvLEA18	-1.001	other	thellun_all_rep_c40374_3	**177**	-1.065	other
**17**	At2g33690	PvLEA18	-1.311	other				
**18**	At2g35300	LEA_1	-1.156	other	thellun_all_rep_c15526_6	**108**	-1.287	other
**19**	At2g36640	LEA_4	-1.023	other				
**20**	At2g40170	LEA_5	-1.407	other				
**21**	At2g41260	AtM	-0.704	secreted				
**22**	At2g41280	AtM	-0.011	secreted	thellun_all_c9151_1	93	0.001	secreted
**23**	At2g42530	LEA_4	-0.542	Chloroplast	thellun_all_rep_c1044_2	**55**	-0.594	Chloroplast
					thellun_all_rep_c26948_3	145	-0.757	Mitochondrion
					thellun_all_rep_c29067_1	157	-0.361	Chloroplast
					cluster504_3	**198**	-0.414	Chloroplast
**24**	At2g42540	LEA_4	-0.554	Chloroplast	thellun_all_rep_c36160_3	168	-1.07	other
**25**	At2g42560	LEA_4	-0.978	other	thellun_all_c26853_3	144	-1.347	other
**26**	At2g44060	LEA_2	-0.314	other	thellun_all_rep_c2225_3	63	0.141	other
					thellun_all_rep_c26673_2	142	0.239	secreted
**27**	At2g46140	LEA_2	0.123	other	thellun_all_rep_c2057_1	**60**	0.057	other
**28**	At3g02480	LEA_4	-1.213	other				
**29**	At3g15670	LEA_4	-1.369	other	thellun_all_rep_c7426_3	84	-1.604	other
**30**	At3g17520	LEA_4	-1.047	secreted	thellun_all_rep_c3347_2	**64**	-1.177	secreted
**31**	At3g22490	SMP	-0.193	other	thellun_all_c27216_3	148	0.334	other
**32**	At3g22500	SMP	-0.341	other	thellun_all_c27216_1	146	-0.619	other
**33**	At3g50970	dehydrin	-1.173	other				
**34**	At3g50980	dehydrin	-1.053	other	thellun_all_c7357_1	80	-0.898	Chloroplast
**35**	At3g51810	LEA_5	-1.468	other				
**36**	At3g53040	LEA_4	-1.194	other	thellun_all_c32122_2	160	-0.848	other
**37**	At3g53770	LEA_3	-0.79	Mitochondrion	thellun_all_rep_c2197_2	62	-0.803	Mitochondrion
**38**	At4g02380	LEA_3	-0.36	Chloroplast	thellun_all_rep_c246_1	**52**	-0.466	Chloroplast

**39**	At4g13230	LEA_4	-0.831	Mitochondrion				
**40**	At4g13560	LEA_4	-1.181	other	thellun_all_c7405_1	**81**	-1.183	other
**41**	At4g15910	LEA_3	-0.526	Chloroplast	thellun_all_rep_c863_2	**54**	-0.451	Chloroplast
**42**	At4g21020	LEA_4	-1.291	Mitochondrion	thellun_all_c8752_1	90	-1.773	other
**43**	At4g36600	LEA_4	-1.072	Mitochondrion				
**44**	At4g38410	dehydrin	-1.629	other	thellun_all_c14197_2	103	-1.156	other
**45**	At4g39130	dehydrin	-0.774	other				
**46**	At5g06760	LEA_1	-0.815	other	thellun_all_rep_c5248_2	**72**	-0.81	other
**47**	At5g27980	SMP	-0.373	other	thellun_all_c34179_3	165	-0.864	other
**48**	At5g44310	LEA_4	-1.409	Chloroplast	thellun_all_c38897_1	175	-0.914	Mitochondrion
**49**	At5g53260	SMP	-0.273	Chloroplast				
**50**	At5g53270	SMP	-0.103	other				
**51**	At5g66400	dehydrin	-1.182	other	thellun_all_rep_c1101_3	**57**	-1.018	other
					thellun_all_rep_c25754_3	136	-1.396	other
					thellun_all_rep_c35985_1	167	-1.221	other
					thellun_all_rep_c36886_2	172	-0.926	other

[1]Protein sequence number as defined in Supplemental Table 2.

[2]The protein family domains, GRAVY (grand average of hydropathy), and predicted subcellular localization for *Arabidopsis* LEA proteins were obtained from Hundertmark and Hincha (2008).

[3]Representative of putative *T. salsuginea* LEA proteins that showed distinct sequence models.
[4]GRAVY quantitates the hydrophilicity of proteins based on amino acid composition using the protparam algorithm (http://web.expasy.org/protparam/). Subcellular localization was predicted from protein sequence analysis using the targetP algorithm.
Empty cells indicate no orthologous protein was found for *T. salsuginea* in the sequence assembly.
Bold numbers indicate complete coding region for *Arabidopsis* and predicted to be complete coding region for *T. salsuginea* based on protein sequence alignment between the two species.
