# Supplementary material for: Transcriptome sequencing and microarray design for functional genomics in the extremophile Arabidopsis relative Thellungiella salsuginea (Eutrema salsugineum)
Source: BMC Genomics. 2013 Nov 14;14:793. doi: 10.1186/1471-2164-14-793 (PMC3832907; doi:10.1186/1471-2164-14-793)
Supplement: Additional file 15 — Number of probes identified from the reciprocal hybridization of RNA samples derived from non-acclimated or cold acclimated leaves to Arabidopsis and T. salsuginea arrays. [file 1471-2164-14-793-S15.pdf]

Additional file 15.

Type of array	*Arabidopsis*	*Arabidopsis*	*Thellungiella*	*Thellungiella*
Hybridized RNA sample[1]	Col-0	Yukon	Col-0	Yukon
Number of probes[2]	37148 ± 5183	21129 ± 3846	43553 ± 1588	45017 ± 166
Differentially expressed probes[3]	623	3336	548	10248
Unique AGI locus	512	2722	321[#]	4425[#]

[1]Source of RNA samples. Altogether 6 hybridizations with RNA derived from leaves of three biological replicates from non-acclimated or cold acclimated plants.
[2]Mean of six hybridizations ± standard deviation. Number of probes with hybridization signal intensities at least 1.5-fold above background.
[3]Differentially expressed probes were identified using a linear model in the LIMMA package and false discovery rate ($p < 0.05$) controlled by Benjamini and Hochberg (1995) method implemented in the Robin software.
[#]Orthologous genes were identified based on protein sequence similarity search using BLASTX, $E < 10^{-10}$.
