# Supplementary material for: Transcriptome sequencing and microarray design for functional genomics in the extremophile Arabidopsis relative Thellungiella salsuginea (Eutrema salsugineum)
Source: BMC Genomics. 2013 Nov 14;14:793. doi: 10.1186/1471-2164-14-793 (PMC3832907; doi:10.1186/1471-2164-14-793)

Additional file 5

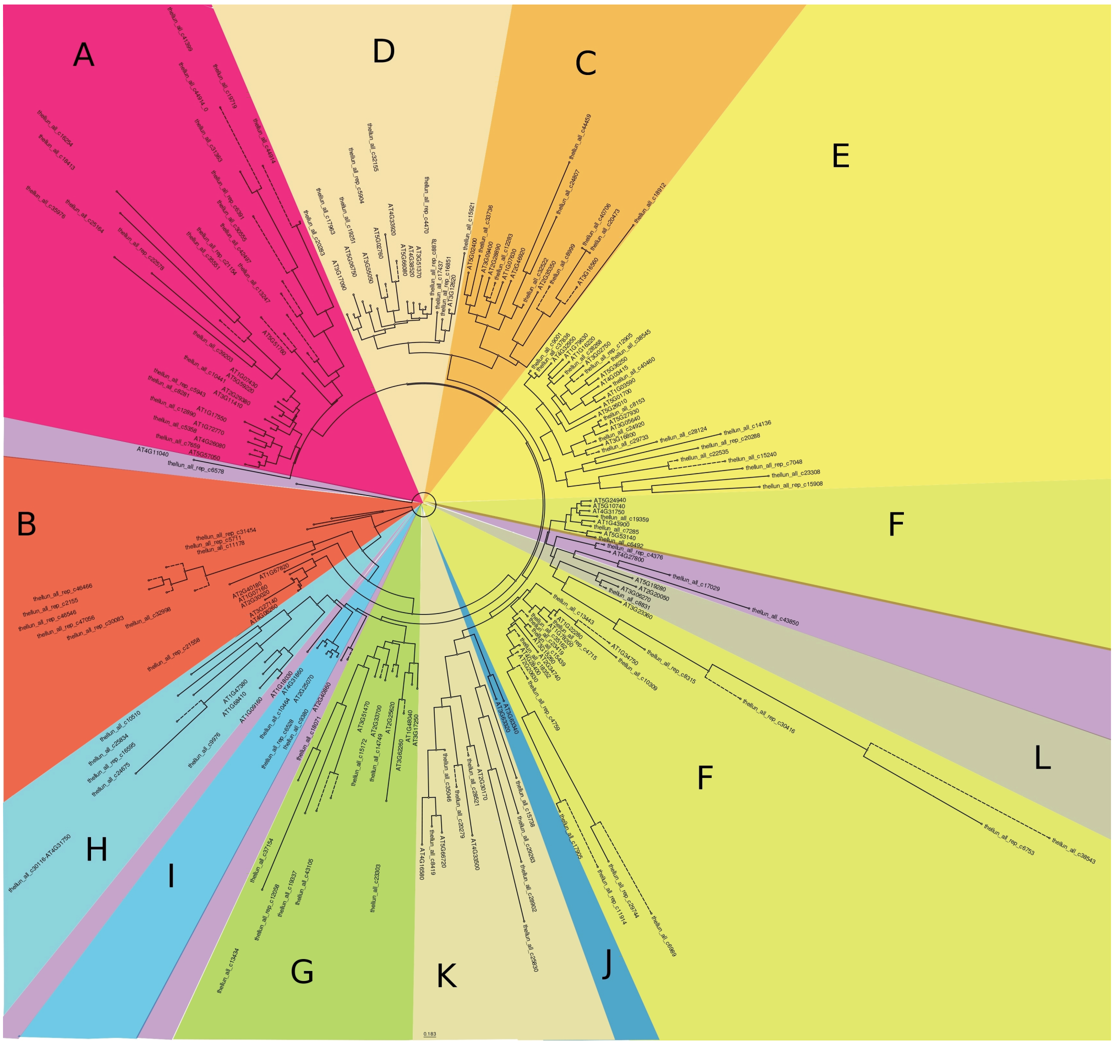

Supplement: Additional file 5 — Unrooted phylogenetic tree of all Arabidopsis and T. salsuginea PP2C proteins shown in Additional file3. [file 1471-2164-14-793-S5.pdf]
