# Supplementary material for: Transcriptome sequencing and microarray design for functional genomics in the extremophile Arabidopsis relative Thellungiella salsuginea (Eutrema salsugineum)
Source: BMC Genomics. 2013 Nov 14;14:793. doi: 10.1186/1471-2164-14-793 (PMC3832907; doi:10.1186/1471-2164-14-793)
Supplement: Additional file 16 — Statistical analysis showing over- and under-representation of MapMan functional groups among significantly cold regulated genes. [file 1471-2164-14-793-S16.pdf]

Additional file 16.

	Up-regulated				Down-regulated			
	Yukon		Col-0		Yukon		Col-0	
Mapman functional group	Number of genes[1]	*p*-value[2]	Number of genes[1]	*p*-value[2]	Number of genes[1]	*p*-value[2]	Number of genes[1]	*p*-value[2]
Photosynthesis	12	***3.46E-041***	1	6.53E-001	502	**1.49E-129**	13	**1.78E-004**
Major CHO metabolism	24	9.90E-002	9	**1.55E-004**	23	5.10E-001	2	5.35E-001
Minor CHO metabolism	21	7.61E-001	3	3.82E-001	14	1.35E-001	1	1.00E+000
Glycolysis	51	**4.45E-012**	2	3.51E-001	19	9.46E-001	1	7.34E-001
Fermentation	11	**1.46E-002**	3	**9.67E-003**	5	1.00E+000	ND	NA
Gluconeogenese/ glyoxylate cycle	6	7.87E-002	ND	NA	2	1.00E+000	ND	NA
Oxidative pentose phosphate cycle	11	1.39E-001	ND	NA	13	7.61E-002	ND	NA
TCA / organic acids transformation	22	2.74E-001	2	3.77E-001	45	**7.03E-007**	8	**1.78E-004**
Mitochondrial electron transport	16	***4.08E-002***	1	1.00E+000	12	***1.82E-005***	1	1.00E+000
Cell wall	60	5.13E-001	6	7.53E-001	160	**1.38E-026**	3	6.49E-001
Lipid metabolism	70	8.40E-001	6	6.06E-001	133	**2.92E-007**	6	6.34E-001
Nitrogen metabolism	10	5.85E-001	2	1.57E-001	24	**5.30E-005**	2	1.79E-001
Amino acid metabolism	60	7.84E-001	4	6.76E-001	115	**4.34E-007**	3	1.00E+000
Sulfur assimilation	ND	NA	ND	NA	12	**1.56E-004**	ND	NA
Metal handling	32	**5.06E-004**	ND	NA	14	4.37E-001	ND	NA
Secondary metabolism	62	1.02E-001	10	1.26E-001	80	**1.82E-002**	4	1.00E+000
Hormone metabolism	62	1.00E+000	3	6.35E-001	152	**4.45E-015**	19	**1.19E-004**
Cofactor and vitamine metabolism	13	8.32E-001	ND	NA	9	3.04E-001	1	6.09E-001
Tetrapyrrole synthesis	2	***1.97E-002***	ND	NA	35	**5.31E-008**	2	2.61E-001
Stress	212	**6.16E-012**	10	9.64E-001	191	**2.08E-002**	5	1.79E-001
Redox regulation	43	7.23E-001	1	8.18E-001	60	1.00E+000	5	2.61E-001
Polyamine metabolism	13	**7.68E-007**	ND	NA	ND	NA	ND	NA
Nucleotide metabolism	27	6.90E-001	2	7.73E-001	20	1.73E-001	1	1.00E+000
Biodegradation of Xenobiotics	18	**1.58E-007**	ND	NA	2	4.04E-001	ND	NA
C1 metabolism	6	1.00E+000	ND	NA	8	9.70E-001	1	5.38E-001
Misc	247	**5.57E-005**	37	**2.26E-005**	392	**2.06E-025**	26	5.62E-002
RNA	350	4.81E-001	18	1.27E-001	303	***1.65E-008***	26	6.96E-001
DNA	34	***4.63E-005***	3	***2.88E-002***	42	***9.46E-007***	1	***1.27E-003***
Protein	552	7.55E-001	25	1.08E-001	375	***1.32E-049***	30	2.98E-001
Signalling	162	6.04E-001	11	9.64E-001	177	5.93E-001	16	5.05E-001
Cell	92	3.14E-001	5	6.04E-001	130	9.77E-001	4	3.86E-001
Micro RNA, natural antisense, etc	ND	NA	2	1.00E+000	ND	NA	3	6.34E-001
Development	109	**2.42E-002**	6	1.00E+000	83	7.04E-002	2	1.81E-001
Transport	164	**1.97E-002**	21	**2.34E-002**	179	3.70E-001	8	6.68E-001
Not assigned	2016	***5.01E-007***	100	3.06E-001	2379	***3.45E-021***	117	9.75E-001

[1]Number of genes was found in each significant gene lists identified from the respective RNA hybridized to species-specific arrays.

[2]*p*-value from Fisher's exact test with false discovery rate controlled by Benjamini and Hochberg (1995) method. *P*-values highlighted in bold and italic were significant ($p < 0.05$) for the proportion of genes in a functional group that was over- or under-represented, respectively, in the respective gene list compared to all corresponding genes represented on the array.

ND, not detected; NA, not available.
